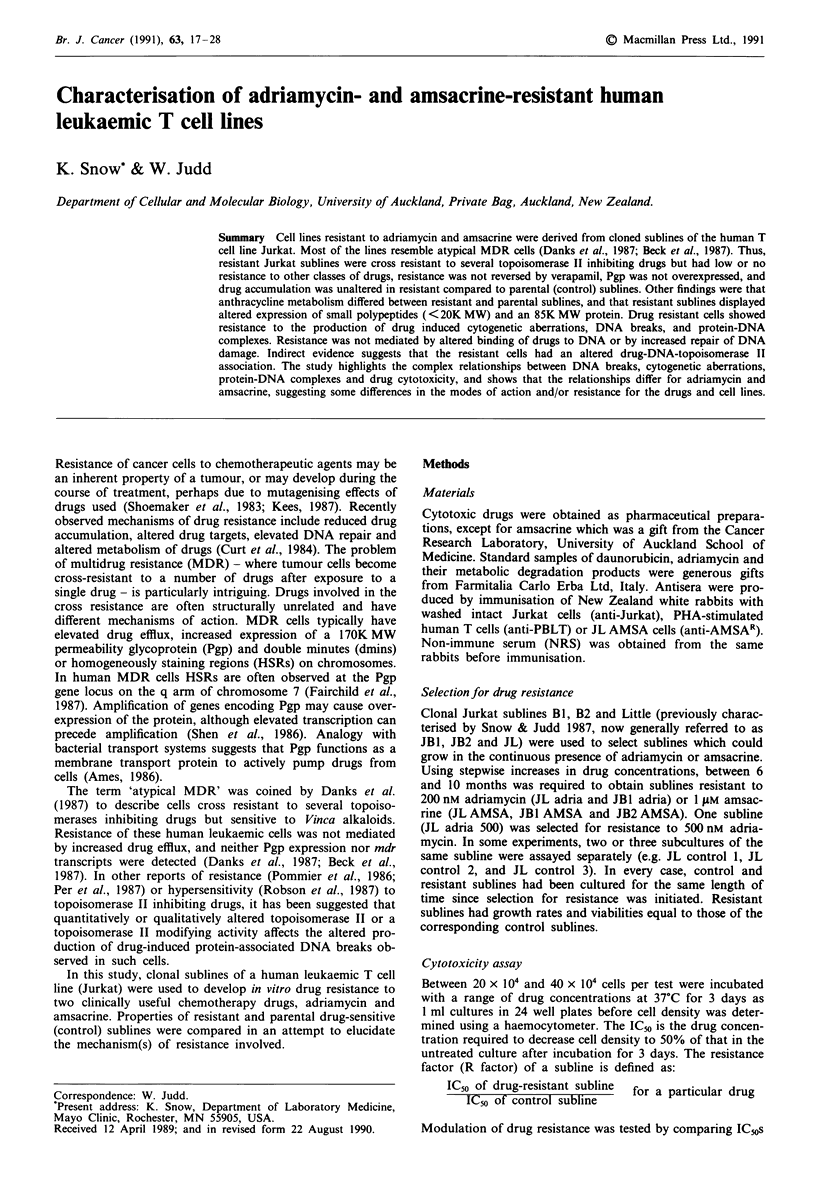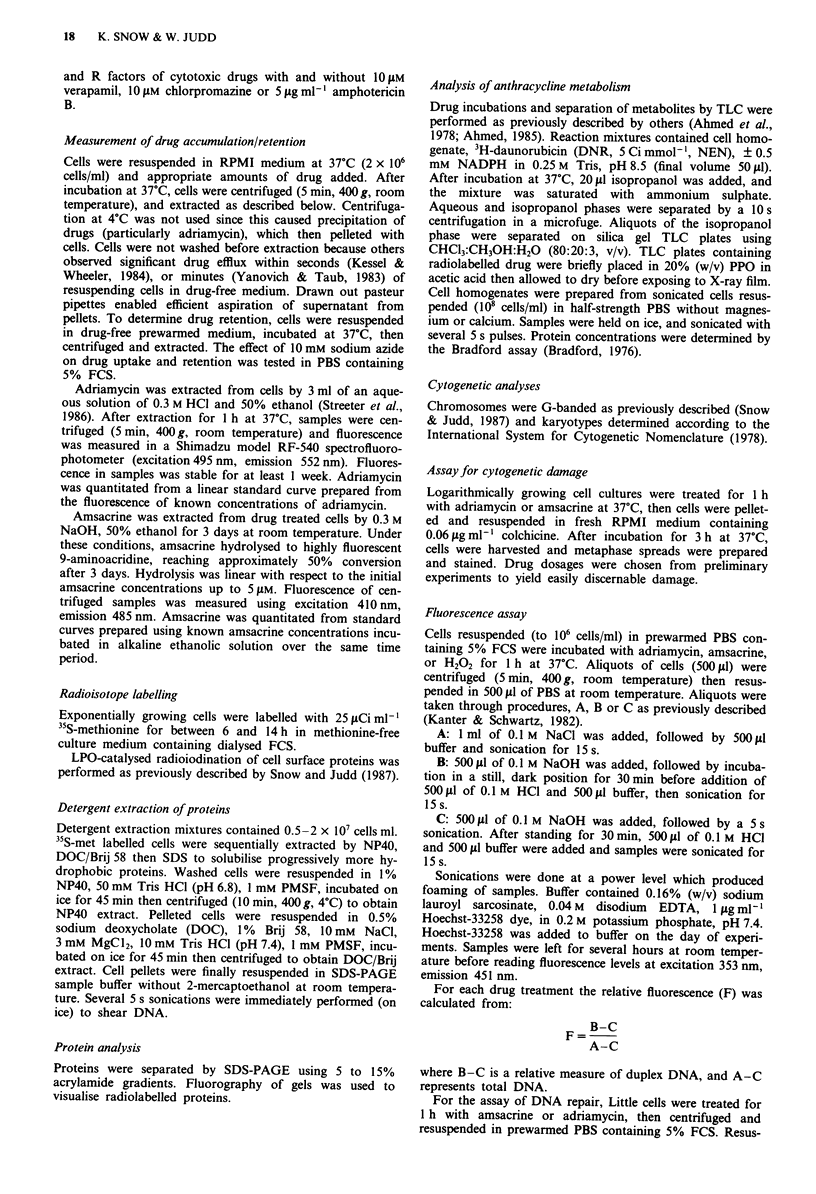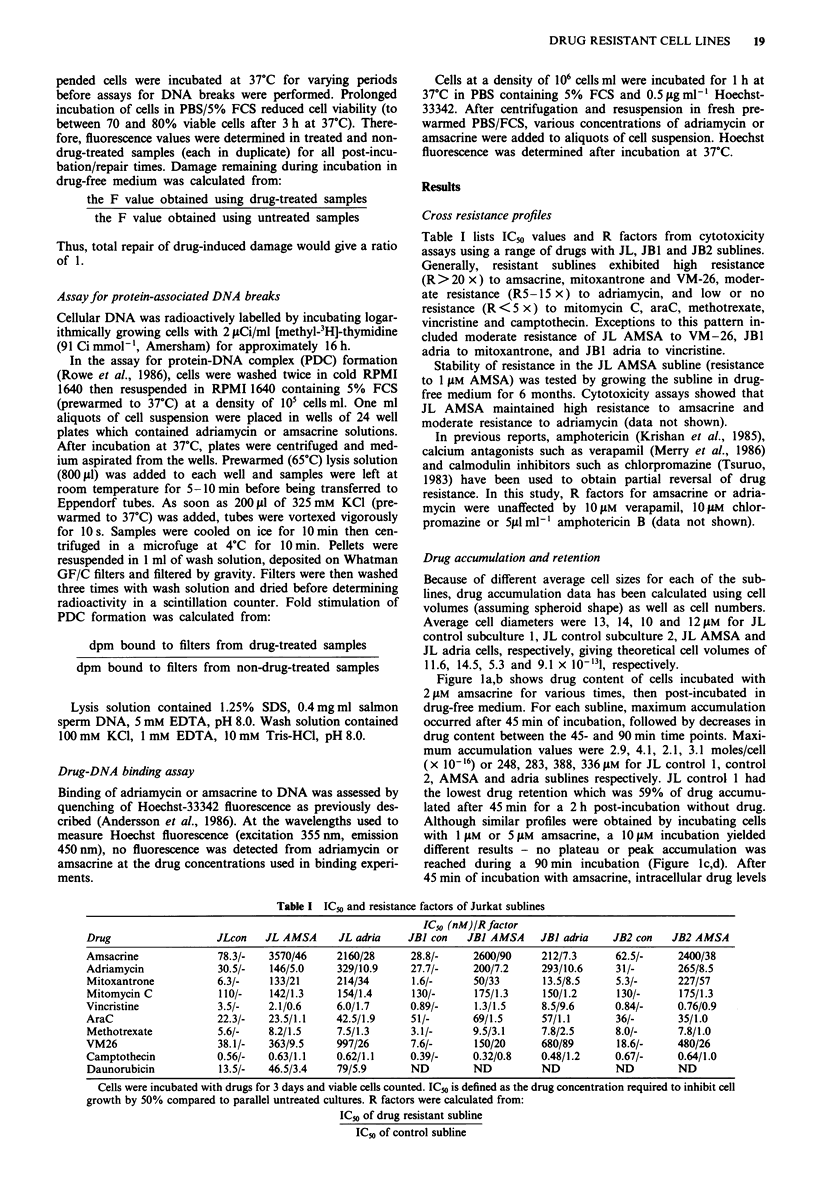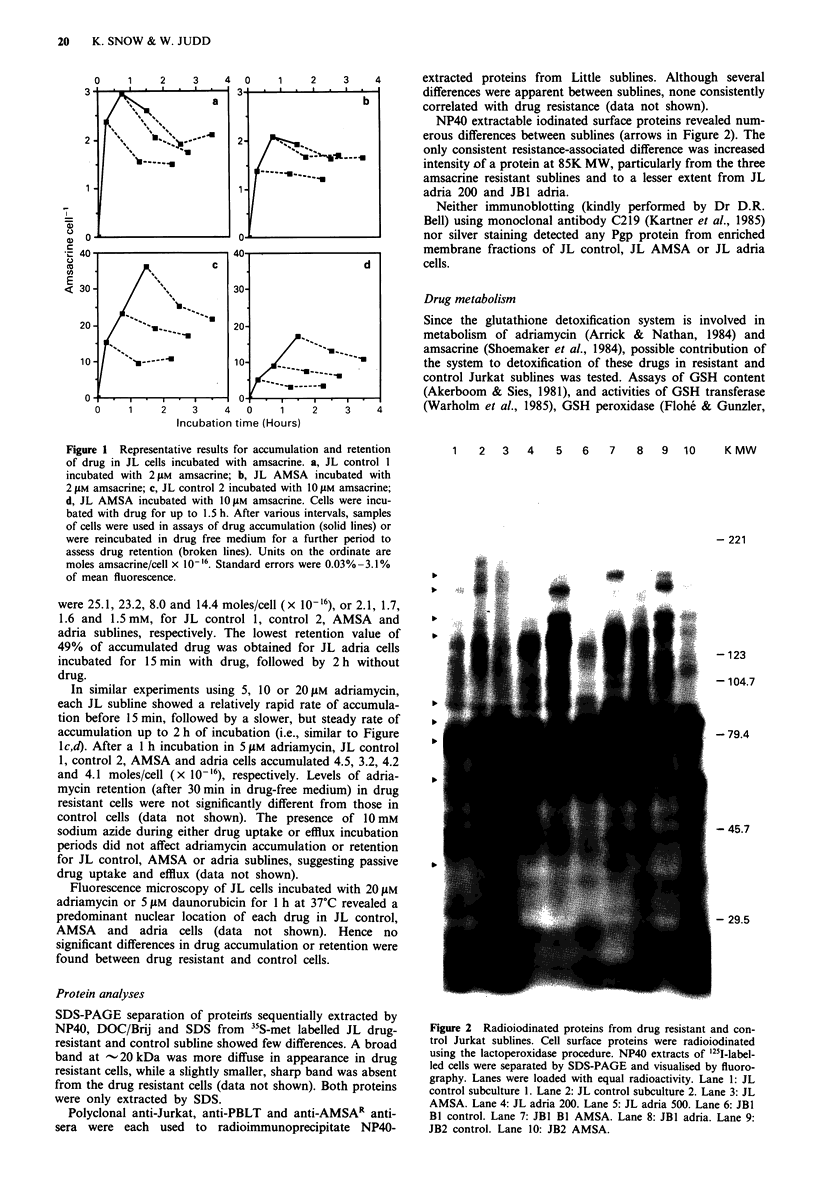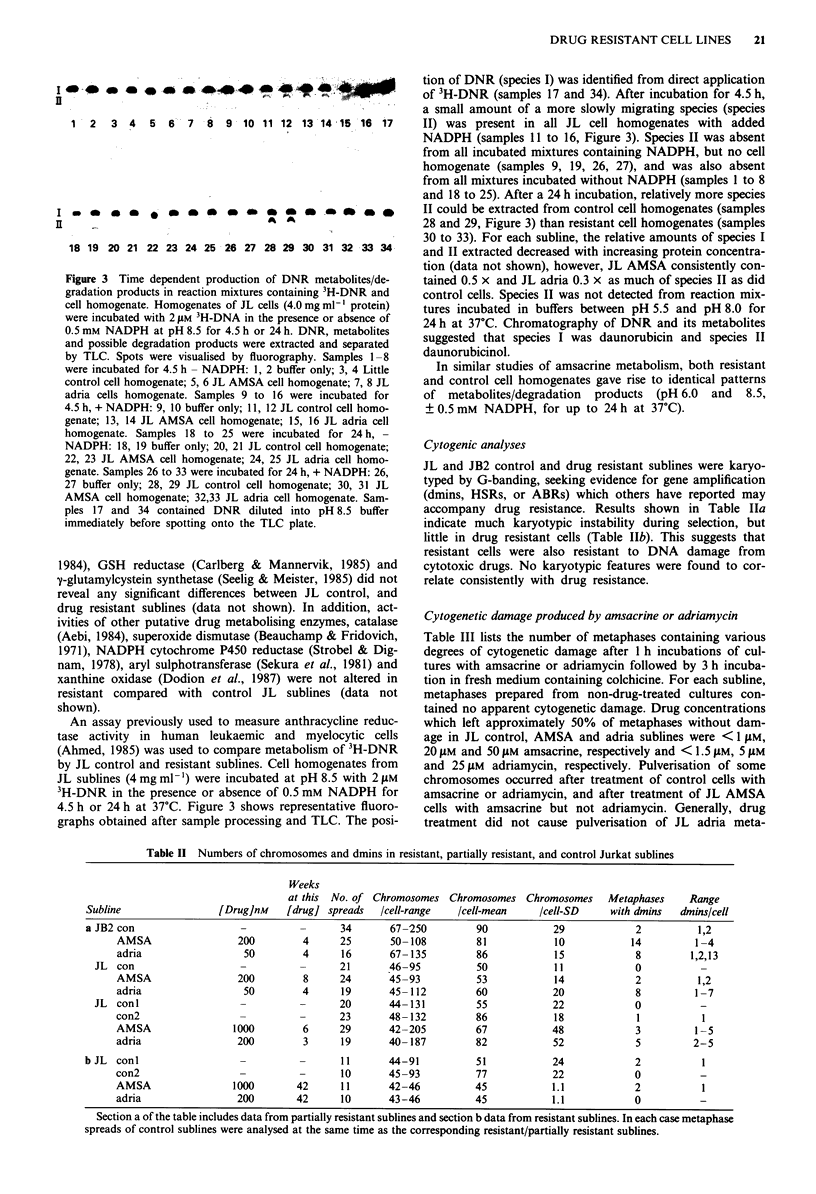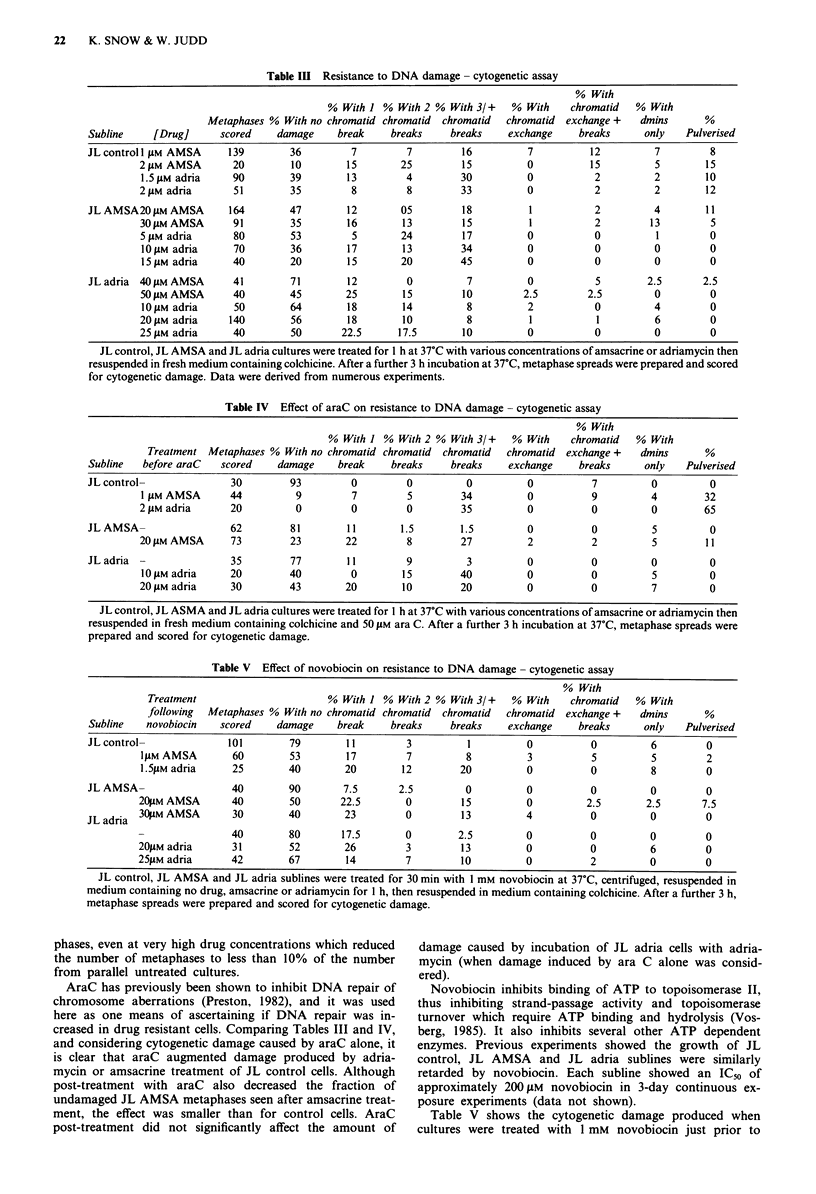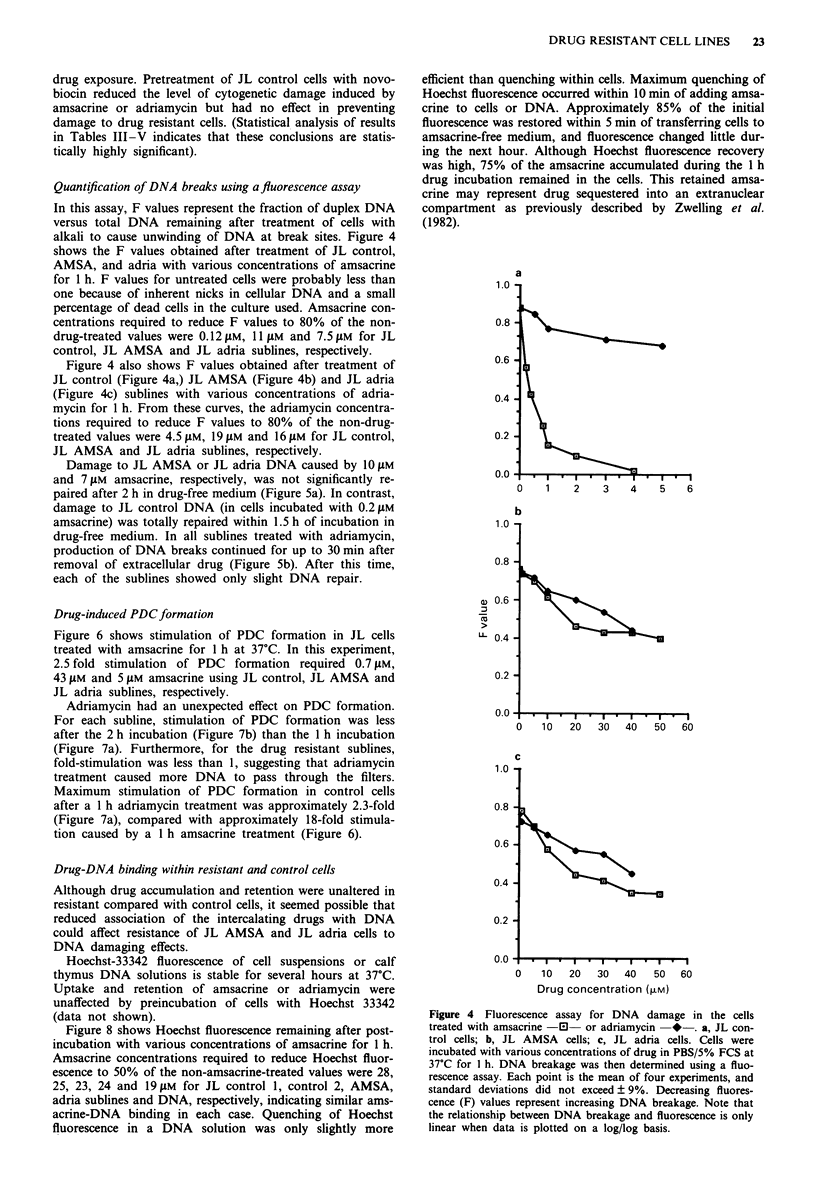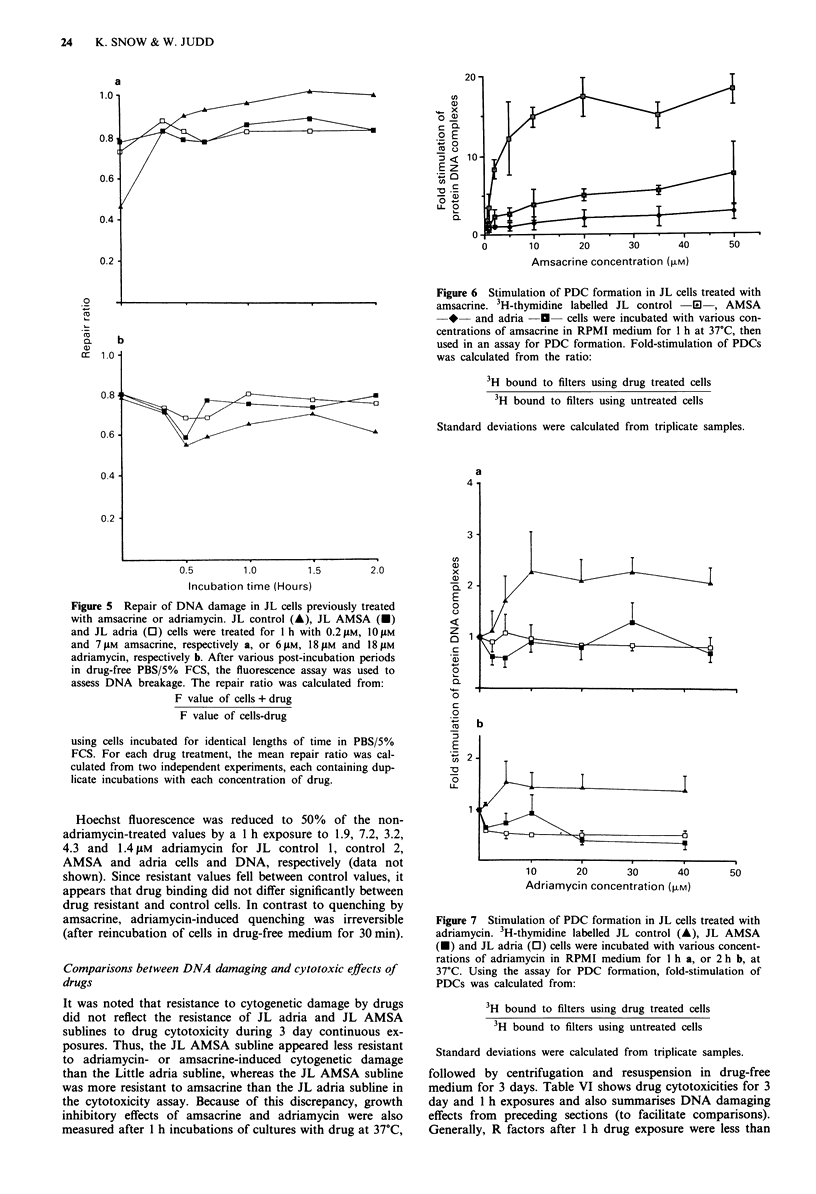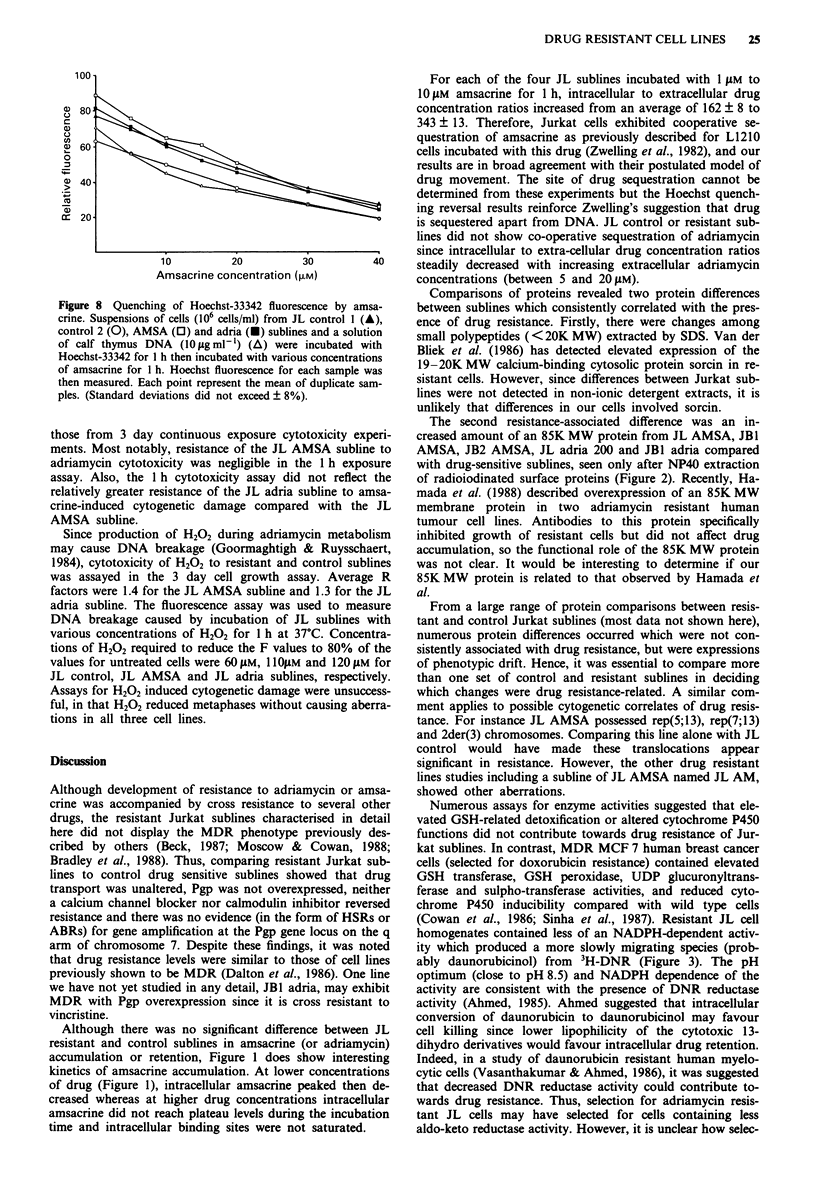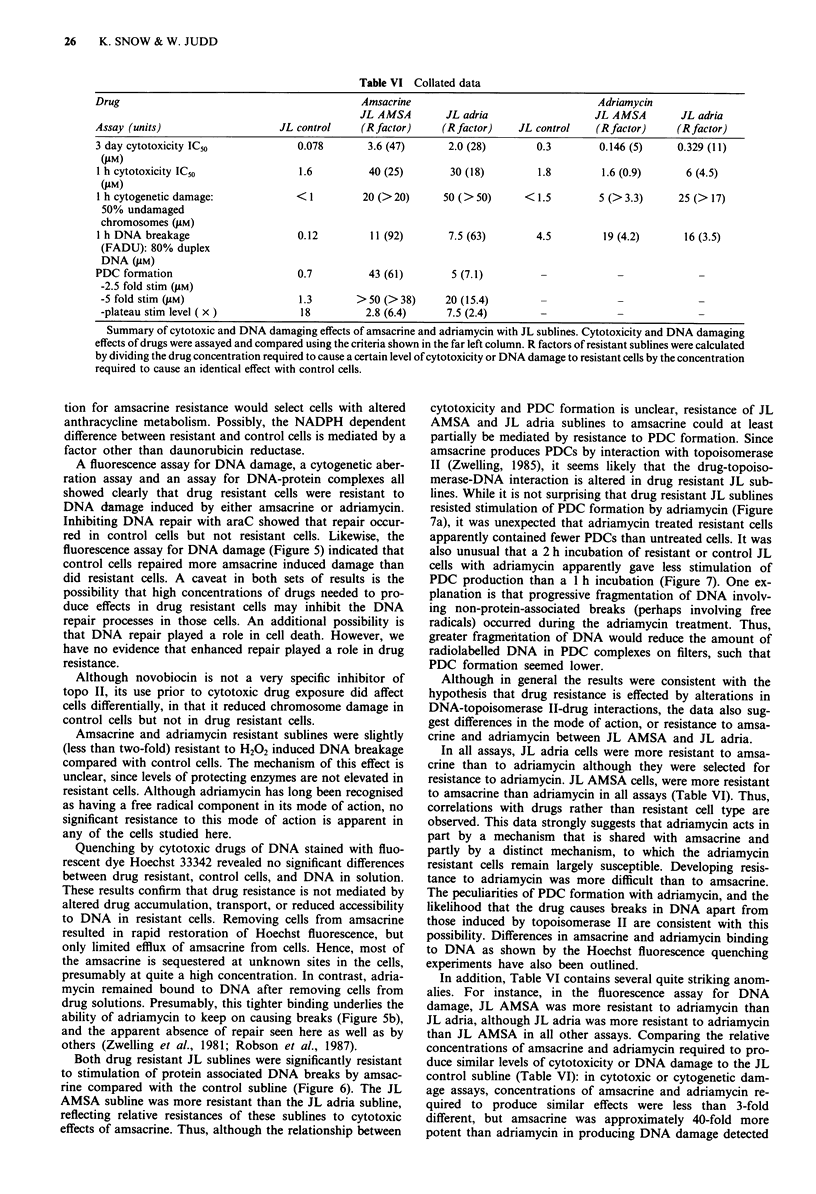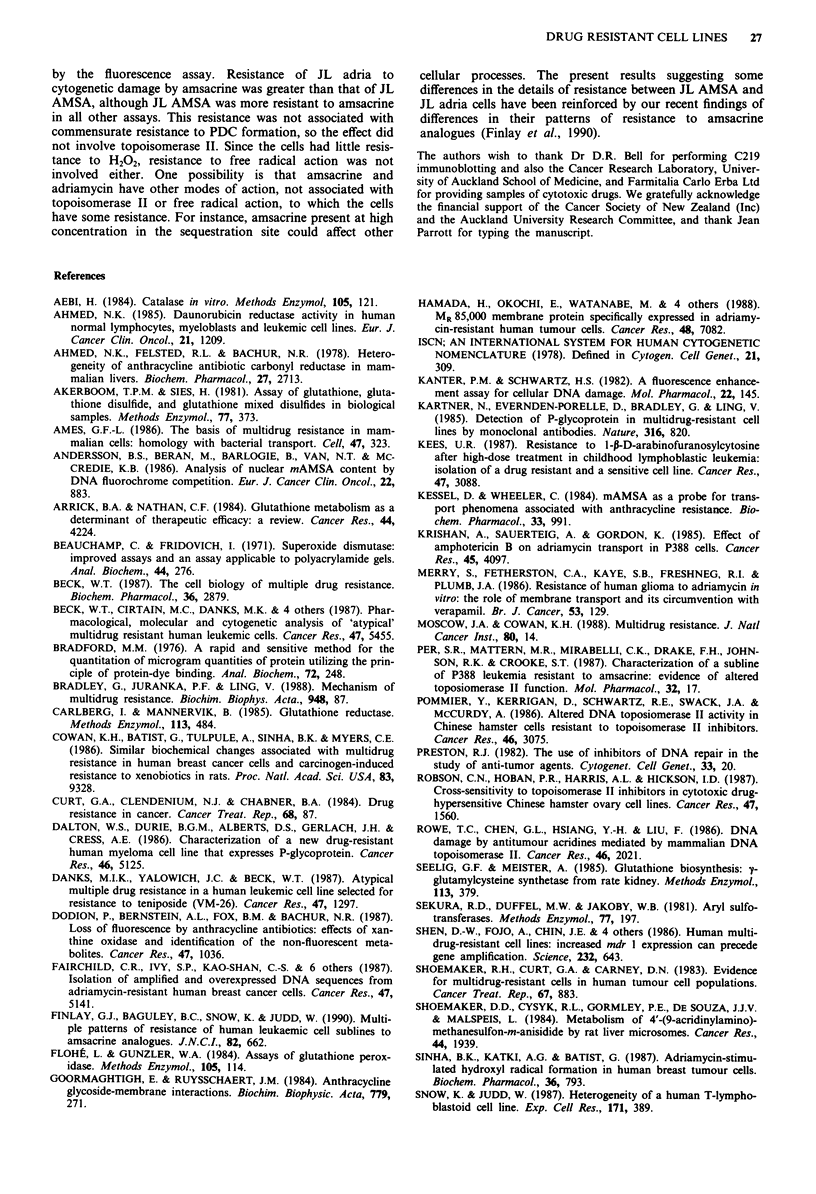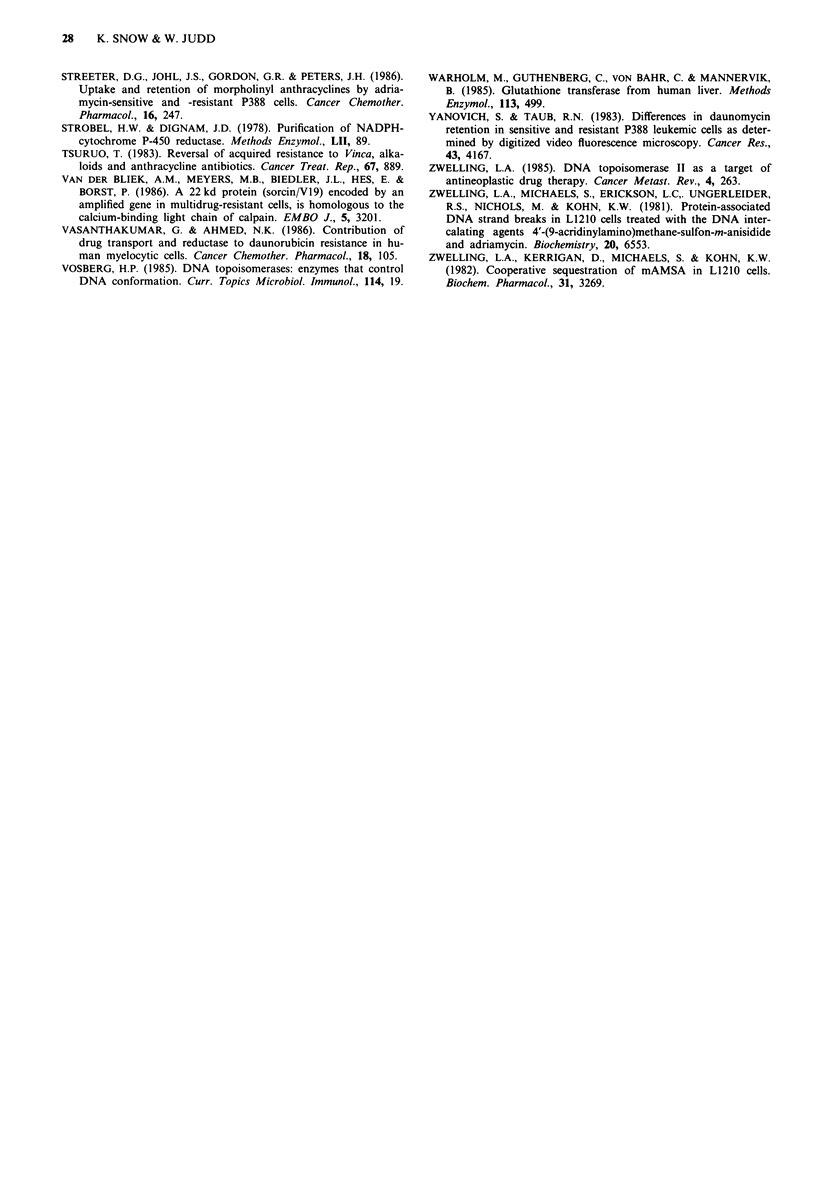# Characterisation of adriamycin- and amsacrine-resistant human leukaemic T cell lines.

**DOI:** 10.1038/bjc.1991.7

**Published:** 1991-01

**Authors:** K. Snow, W. Judd

**Affiliations:** Department of Cellular and Molecular Biology, University of Auckland, New Zealand.

## Abstract

**Images:**


Br. J. Cancer (1991), 63, 17-28 © Macmillan Press Ltd., 1991

### Characterisation of adriamycin- and amsacrine-resistant human leukaemic T cell lines

K. Snow* & W. Judd

*Department of Cellular and Molecular Biology, University of Auckland, Private Bag, Auckland, New Zealand.*

**Summary** Cell lines resistant to adriamycin and amsacrine were derived from cloned sublines of the human T cell line Jurkat. Most of the lines resemble atypical MDR cells (Danks *et al.*, 1987; Beck *et al.*, 1987). Thus, resistant Jurkat sublines were cross resistant to several topoisomerase II inhibiting drugs but had low or no resistance to other classes of drugs, resistance was not reversed by verapamil, Pgp was not overexpressed, and drug accumulation was unaltered in resistant compared to parental (control) sublines. Other findings were that anthracycline metabolism differed between resistant and parental sublines, and that resistant sublines displayed altered expression of small polypeptides (<20K MW) and an 85K MW protein. Drug resistant cells showed resistance to the production of drug induced cytogenetic aberrations, DNA breaks, and protein-DNA complexes. Resistance was not mediated by altered binding of drugs to DNA or by increased repair of DNA damage. Indirect evidence suggests that the resistant cells had an altered drug-DNA-topoisomerase II association. The study highlights the complex relationships between DNA breaks, cytogenetic aberrations, protein-DNA complexes and drug cytotoxicity, and shows that the relationships differ for adriamycin and amsacrine, suggesting some differences in the modes of action and/or resistance for the drugs and cell lines.

Resistance of cancer cells to chemotherapeutic agents may be an inherent property of a tumour, or may develop during the course of treatment, perhaps due to mutagenising effects of drugs used (Shoemaker *et al.*, 1983; Kees, 1987). Recently observed mechanisms of drug resistance include reduced drug accumulation, altered drug targets, elevated DNA repair and altered metabolism of drugs (Curt *et al.*, 1984). The problem of multidrug resistance (MDR) – where tumour cells become cross-resistant to a number of drugs after exposure to a single drug – is particularly intriguing. Drugs involved in the cross resistance are often structurally unrelated and have different mechanisms of action. MDR cells typically have elevated drug efflux, increased expression of a 170K MW permeability glycoprotein (Pgp) and double minutes (dmins) or homogeneously staining regions (HSRs) on chromosomes. In human MDR cells HSRs are often observed at the Pgp gene locus on the q arm of chromosome 7 (Fairchild *et al.*, 1987). Amplification of genes encoding Pgp may cause overexpression of the protein, although elevated transcription can precede amplification (Shen *et al.*, 1986). Analogy with bacterial transport systems suggests that Pgp functions as a membrane transport protein to actively pump drugs from cells (Ames, 1986).

The term 'atypical MDR' was coined by Danks *et al.* (1987) to describe cells cross resistant to several topoisomerases inhibiting drugs but sensitive to *Vinca* alkaloids. Resistance of these human leukaemic cells was not mediated by increased drug efflux, and neither Pgp expression nor *mdr* transcripts were detected (Danks *et al.*, 1987; Beck *et al.*, 1987). In other reports of resistance (Pommier *et al.*, 1986; Per *et al.*, 1987) or hypersensitivity (Robson *et al.*, 1987) to topoisomerase II inhibiting drugs, it has been suggested that quantitatively or qualitatively altered topoisomerase II or a topoisomerase II modifying activity affects the altered production of drug-induced protein-associated DNA breaks observed in such cells.

In this study, clonal sublines of a human leukaemic T cell line (Jurkat) were used to develop *in vitro* drug resistance to two clinically useful chemotherapy drugs, adriamycin and amsacrine. Properties of resistant and parental drug-sensitive (control) sublines were compared in an attempt to elucidate the mechanism(s) of resistance involved.

Correspondence: W. Judd.
*Present address: K. Snow, Department of Laboratory Medicine, Mayo Clinic, Rochester, MN 55905, USA.
Received 12 April 1989; and in revised form 22 August 1990.

### Methods

### *Materials*

Cytotoxic drugs were obtained as pharmaceutical preparations, except for amsacrine which was a gift from the Cancer Research Laboratory, University of Auckland School of Medicine. Standard samples of daunorubicin, adriamycin and their metabolic degradation products were generous gifts from Farmitalia Carlo Erba Ltd, Italy. Antisera were produced by immunisation of New Zealand white rabbits with washed intact Jurkat cells (anti-Jurkat), PHA-stimulated human T cells (anti-PBLT) or JL AMSA cells (anti-AMSA$^R$). Non-immune serum (NRS) was obtained from the same rabbits before immunisation.

### *Selection for drug resistance*

Clonal Jurkat sublines B1, B2 and Little (previously characterised by Snow & Judd 1987, now generally referred to as JB1, JB2 and JL) were used to select sublines which could grow in the continuous presence of adriamycin or amsacrine. Using stepwise increases in drug concentrations, between 6 and 10 months was required to obtain sublines resistant to 200 nM adriamycin (JL adria and JB1 adria) or 1 μM amsacrine (JL AMSA, JB1 AMSA and JB2 AMSA). One subline (JL adria 500) was selected for resistance to 500 nM adriamycin. In some experiments, two or three subcultures of the same subline were assayed separately (e.g. JL control 1, JL control 2, and JL control 3). In every case, control and resistant sublines had been cultured for the same length of time since selection for resistance was initiated. Resistant sublines had growth rates and viabilities equal to those of the corresponding control sublines.

### *Cytotoxicity assay*

Between $20 \times 10^4$ and $40 \times 10^4$ cells per test were incubated with a range of drug concentrations at 37°C for 3 days as 1 ml cultures in 24 well plates before cell density was determined using a haemocytometer. The $IC_{50}$ is the drug concentration required to decrease cell density to 50% of that in the untreated culture after incubation for 3 days. The resistance factor (R factor) of a subline is defined as:

$$\frac{IC_{50} \text{ of drug-resistant subline}}{IC_{50} \text{ of control subline}} \quad \text{for a particular drug}$$

Modulation of drug resistance was tested by comparing $IC_{50}$s and R factors of cytotoxic drugs with and without 10 μM verapamil, 10 μM chlorpromazine or 5 μg $ml^{-1}$ amphotericin B.

*Measurement of drug accumulation/retention*

Cells were resuspended in RPMI medium at 37°C ($2 \times 10^6$ cells/ml) and appropriate amounts of drug added. After incubation at 37°C, cells were centrifuged (5 min, 400 *g*, room temperature), and extracted as described below. Centrifugation at 4°C was not used since this caused precipitation of drugs (particularly adriamycin), which then pelleted with cells. Cells were not washed before extraction because others observed significant drug efflux within seconds (Kessel & Wheeler, 1984), or minutes (Yanovich & Taub, 1983) of resuspending cells in drug-free medium. Drawn out pasteur pipettes enabled efficient aspiration of supernatant from pellets. To determine drug retention, cells were resuspended in drug-free prewarmed medium, incubated at 37°C, then centrifuged and extracted. The effect of 10 mM sodium azide on drug uptake and retention was tested in PBS containing 5% FCS.

Adriamycin was extracted from cells by 3 ml of an aqueous solution of 0.3 M HCl and 50% ethanol (Streeter *et al.*, 1986). After extraction for 1 h at 37°C, samples were centrifuged (5 min, 400 *g*, room temperature) and fluorescence was measured in a Shimadzu model RF-540 spectrofluorophotometer (excitation 495 nm, emission 552 nm). Fluorescence in samples was stable for at least 1 week. Adriamycin was quantitated from a linear standard curve prepared from the fluorescence of known concentrations of adriamycin.

Amsacrine was extracted from drug treated cells by 0.3 M NaOH, 50% ethanol for 3 days at room temperature. Under these conditions, amsacrine hydrolysed to highly fluorescent 9-aminoacridine, reaching approximately 50% conversion after 3 days. Hydrolysis was linear with respect to the initial amsacrine concentrations up to 5 μM. Fluorescence of centrifuged samples was measured using excitation 410 nm, emission 485 nm. Amsacrine was quantitated from standard curves prepared using known amsacrine concentrations incubated in alkaline ethanolic solution over the same time period.

*Radioisotope labelling*

Exponentially growing cells were labelled with 25 μCi $ml^{-1}$ $^{35}$S-methionine for between 6 and 14 h in methionine-free culture medium containing dialysed FCS.

LPO-catalysed radioiodination of cell surface proteins was performed as previously described by Snow and Judd (1987).

*Detergent extraction of proteins*

Detergent extraction mixtures contained $0.5-2 \times 10^7$ cells ml. $^{35}$S-met labelled cells were sequentially extracted by NP40, DOC/Brij 58 then SDS to solubilise progressively more hydrophobic proteins. Washed cells were resuspended in 1% NP40, 50 mM Tris HCl (pH 6.8), 1 mM PMSF, incubated on ice for 45 min then centrifuged (10 min, 400 *g*, 4°C) to obtain NP40 extract. Pelleted cells were resuspended in 0.5% sodium deoxycholate (DOC), 1% Brij 58, 10 mM NaCl, 3 mM $MgCl_2$, 10 mM Tris HCl (pH 7.4), 1 mM PMSF, incubated on ice for 45 min then centrifuged to obtain DOC/Brij extract. Cell pellets were finally resuspended in SDS-PAGE sample buffer without 2-mercaptoethanol at room temperature. Several 5 s sonications were immediately performed (on ice) to shear DNA.

*Protein analysis*

Proteins were separated by SDS-PAGE using 5 to 15% acrylamide gradients. Fluorography of gels was used to visualise radiolabelled proteins.

*Analysis of anthracycline metabolism*

Drug incubations and separation of metabolites by TLC were performed as previously described by others (Ahmed *et al.*, 1978; Ahmed, 1985). Reaction mixtures contained cell homogenate, $^3$H-daunorubicin (DNR, 5 Ci $mmol^{-1}$, NEN), ± 0.5 mM NADPH in 0.25 M Tris, pH 8.5 (final volume 50 μl). After incubation at 37°C, 20 μl isopropanol was added, and the mixture was saturated with ammonium sulphate. Aqueous and isopropanol phases were separated by a 10 s centrifugation in a microfuge. Aliquots of the isopropanol phase were separated on silica gel TLC plates using $CHCl_3$:$CH_3OH$:$H_2O$ (80:20:3, v/v). TLC plates containing radiolabelled drug were briefly placed in 20% (w/v) PPO in acetic acid then allowed to dry before exposing to X-ray film. Cell homogenates were prepared from sonicated cells resuspended ($10^8$ cells/ml) in half-strength PBS without magnesium or calcium. Samples were held on ice, and sonicated with several 5 s pulses. Protein concentrations were determined by the Bradford assay (Bradford, 1976).

*Cytogenetic analyses*

Chromosomes were G-banded as previously described (Snow & Judd, 1987) and karyotypes determined according to the International System for Cytogenetic Nomenclature (1978).

*Assay for cytogenetic damage*

Logarithmically growing cell cultures were treated for 1 h with adriamycin or amsacrine at 37°C, then cells were pelleted and resuspended in fresh RPMI medium containing 0.06 μg $ml^{-1}$ colchicine. After incubation for 3 h at 37°C, cells were harvested and metaphase spreads were prepared and stained. Drug dosages were chosen from preliminary experiments to yield easily discernable damage.

*Fluorescence assay*

Cells resuspended (to $10^6$ cells/ml) in prewarmed PBS containing 5% FCS were incubated with adriamycin, amsacrine, or $H_2O_2$ for 1 h at 37°C. Aliquots of cells (500 μl) were centrifuged (5 min, 400 *g*, room temperature) then resuspended in 500 μl of PBS at room temperature. Aliquots were taken through procedures, A, B or C as previously described (Kanter & Schwartz, 1982).

A: 1 ml of 0.1 M NaCl was added, followed by 500 μl buffer and sonication for 15 s.

B: 500 μl of 0.1 M NaOH was added, followed by incubation in a still, dark position for 30 min before addition of 500 μl of 0.1 M HCl and 500 μl buffer, then sonication for 15 s.

C: 500 μl of 0.1 M NaOH was added, followed by a 5 s sonication. After standing for 30 min, 500 μl of 0.1 M HCl and 500 μl buffer were added and samples were sonicated for 15 s.

Sonications were done at a power level which produced foaming of samples. Buffer contained 0.16% (w/v) sodium lauroyl sarcosinate, 0.04 M disodium EDTA, 1 μg $ml^{-1}$ Hoechst-33258 dye, in 0.2 M potassium phosphate, pH 7.4. Hoechst-33258 was added to buffer on the day of experiments. Samples were left for several hours at room temperature before reading fluorescence levels at excitation 353 nm, emission 451 nm.

For each drug treatment the relative fluorescence (F) was calculated from:

$$F = \frac{B-C}{A-C}$$

where B−C is a relative measure of duplex DNA, and A−C represents total DNA.

For the assay of DNA repair, Little cells were treated for 1 h with amsacrine or adriamycin, then centrifuged and resuspended in prewarmed PBS containing 5% FCS. Resuspended cells were incubated at 37°C for varying periods before assays for DNA breaks were performed. Prolonged incubation of cells in PBS/5% FCS reduced cell viability (to between 70 and 80% viable cells after 3 h at 37°C). Therefore, fluorescence values were determined in treated and non-drug-treated samples (each in duplicate) for all post-incubation/repair times. Damage remaining during incubation in drug-free medium was calculated from:

$$\frac{\text{the F value obtained using drug-treated samples}}{\text{the F value obtained using untreated samples}}$$

Thus, total repair of drug-induced damage would give a ratio of 1.

*Assay for protein-associated DNA breaks*

Cellular DNA was radioactively labelled by incubating logarithmically growing cells with 2 μCi/ml [methyl-$^3$H]-thymidine (91 Ci mmol$^{-1}$, Amersham) for approximately 16 h.

In the assay for protein-DNA complex (PDC) formation (Rowe *et al.*, 1986), cells were washed twice in cold RPMI 1640 then resuspended in RPMI 1640 containing 5% FCS (prewarmed to 37°C) at a density of $10^5$ cells ml. One ml aliquots of cell suspension were placed in wells of 24 well plates which contained adriamycin or amsacrine solutions. After incubation at 37°C, plates were centrifuged and medium aspirated from the wells. Prewarmed (65°C) lysis solution (800 μl) was added to each well and samples were left at room temperature for 5–10 min before being transferred to Eppendorf tubes. As soon as 200 μl of 325 mM KCl (prewarmed to 37°C) was added, tubes were vortexed vigorously for 10 s. Samples were cooled on ice for 10 min then centrifuged in a microfuge at 4°C for 10 min. Pellets were resuspended in 1 ml of wash solution, deposited on Whatman GF/C filters and filtered by gravity. Filters were then washed three times with wash solution and dried before determining radioactivity in a scintillation counter. Fold stimulation of PDC formation was calculated from:

$$\frac{\text{dpm bound to filters from drug-treated samples}}{\text{dpm bound to filters from non-drug-treated samples}}$$

Lysis solution contained 1.25% SDS, 0.4 mg ml salmon sperm DNA, 5 mM EDTA, pH 8.0. Wash solution contained 100 mM KCl, 1 mM EDTA, 10 mM Tris-HCl, pH 8.0.

*Drug-DNA binding assay*

Binding of adriamycin or amsacrine to DNA was assessed by quenching of Hoechst-33342 fluorescence as previously described (Andersson *et al.*, 1986). At the wavelengths used to measure Hoechst fluorescence (excitation 355 nm, emission 450 nm), no fluorescence was detected from adriamycin or amsacrine at the drug concentrations used in binding experiments.

Cells at a density of $10^6$ cells ml were incubated for 1 h at 37°C in PBS containing 5% FCS and 0.5 μg ml$^{-1}$ Hoechst-33342. After centrifugation and resuspension in fresh prewarmed PBS/FCS, various concentrations of adriamycin or amsacrine were added to aliquots of cell suspension. Hoechst fluorescence was determined after incubation at 37°C.

### Results

*Cross resistance profiles*

Table I lists $IC_{50}$ values and R factors from cytotoxicity assays using a range of drugs with JL, JB1 and JB2 sublines. Generally, resistant sublines exhibited high resistance (R > 20 ×) to amsacrine, mitoxantrone and VM-26, moderate resistance (R5–15 ×) to adriamycin, and low or no resistance (R < 5 ×) to mitomycin C, araC, methotrexate, vincristine and camptothecin. Exceptions to this pattern included moderate resistance of JL AMSA to VM-26, JB1 adria to mitoxantrone, and JB1 adria to vincristine.

Stability of resistance in the JL AMSA subline (resistance to 1 μM AMSA) was tested by growing the subline in drug-free medium for 6 months. Cytotoxicity assays showed that JL AMSA maintained high resistance to amsacrine and moderate resistance to adriamycin (data not shown).

In previous reports, amphotericin (Krishan *et al.*, 1985), calcium antagonists such as verapamil (Merry *et al.*, 1986) and calmodulin inhibitors such as chlorpromazine (Tsuruo, 1983) have been used to obtain partial reversal of drug resistance. In this study, R factors for amsacrine or adriamycin were unaffected by 10 μM verapamil, 10 μM chlorpromazine or 5μl ml$^{-1}$ amphotericin B (data not shown).

*Drug accumulation and retention*

Because of different average cell sizes for each of the sublines, drug accumulation data has been calculated using cell volumes (assuming spheroid shape) as well as cell numbers. Average cell diameters were 13, 14, 10 and 12 μM for JL control subculture 1, JL control subculture 2, JL AMSA and JL adria cells, respectively, giving theoretical cell volumes of 11.6, 14.5, 5.3 and 9.1 × $10^{-13}$l, respectively.

Figure 1a,b shows drug content of cells incubated with 2 μM amsacrine for various times, then post-incubated in drug-free medium. For each subline, maximum accumulation occurred after 45 min of incubation, followed by decreases in drug content between the 45- and 90 min time points. Maximum accumulation values were 2.9, 4.1, 2.1, 3.1 moles/cell (× $10^{-16}$) or 248, 283, 388, 336 μM for JL control 1, control 2, AMSA and adria sublines respectively. JL control 1 had the lowest drug retention which was 59% of drug accumulated after 45 min for a 2 h post-incubation without drug. Although similar profiles were obtained by incubating cells with 1 μM or 5 μM amsacrine, a 10 μM incubation yielded different results – no plateau or peak accumulation was reached during a 90 min incubation (Figure 1c,d). After 45 min of incubation with amsacrine, intracellular drug levels

**Table I** $IC_{50}$ and resistance factors of Jurkat sublines

| | | | | $IC_{50}$ (nM)/R factor | | | | |
|---|---|---|---|---|---|---|---|---|
| *Drug* | *JLcon* | *JL AMSA* | *JL adria* | *JB1 con* | *JB1 AMSA* | *JB1 adria* | *JB2 con* | *JB2 AMSA* |
| Amsacrine | 78.3/- | 3570/46 | 2160/28 | 28.8/- | 2600/90 | 212/7.3 | 62.5/- | 2400/38 |
| Adriamycin | 30.5/- | 146/5.0 | 329/10.9 | 27.7/- | 200/7.2 | 293/10.6 | 31/- | 265/8.5 |
| Mitoxantrone | 6.3/- | 133/21 | 214/34 | 1.6/- | 50/33 | 13.5/8.5 | 5.3/- | 227/57 |
| Mitomycin C | 110/- | 142/1.3 | 154/1.4 | 130/- | 175/1.3 | 150/1.2 | 130/- | 175/1.3 |
| Vincristine | 3.5/- | 2.1/0.6 | 6.0/1.7 | 0.89/- | 1.3/1.5 | 8.5/9.6 | 0.84/- | 0.76/0.9 |
| AraC | 22.3/- | 23.5/1.1 | 42.5/1.9 | 51/- | 69/1.5 | 57/1.1 | 36/- | 35/1.0 |
| Methotrexate | 5.6/- | 8.2/1.5 | 7.5/1.3 | 3.1/- | 9.5/3.1 | 7.8/2.5 | 8.0/- | 7.8/1.0 |
| VM26 | 38.1/- | 363/9.5 | 997/26 | 7.6/- | 150/20 | 680/89 | 18.6/- | 480/26 |
| Camptothecin | 0.56/- | 0.63/1.1 | 0.62/1.1 | 0.39/- | 0.32/0.8 | 0.48/1.2 | 0.67/- | 0.64/1.0 |
| Daunorubicin | 13.5/- | 46.5/3.4 | 79/5.9 | ND | ND | ND | ND | ND |

Cells were incubated with drugs for 3 days and viable cells counted. $IC_{50}$ is defined as the drug concentration required to inhibit cell growth by 50% compared to parallel untreated cultures. R factors were calculated from:

$$\frac{IC_{50}\text{ of drug resistant subline}}{IC_{50}\text{ of control subline}}$$

**Figure 1** Representative results for accumulation and retention of drug in JL cells incubated with amsacrine. **a**, JL control 1 incubated with 2 µM amsacrine; **b**, JL AMSA incubated with 2 µM amsacrine; **c**, JL control 2 incubated with 10 µM amsacrine; **d**, JL AMSA incubated with 10 µM amsacrine. Cells were incubated with drug for up to 1.5 h. After various intervals, samples of cells were used in assays of drug accumulation (solid lines) or were reincubated in drug free medium for a further period to assess drug retention (broken lines). Units on the ordinate are moles amsacrine/cell × $10^{-16}$. Standard errors were 0.03%–3.1% of mean fluorescence.

were 25.1, 23.2, 8.0 and 14.4 moles/cell (× $10^{-16}$), or 2.1, 1.7, 1.6 and 1.5 mM, for JL control 1, control 2, AMSA and adria sublines, respectively. The lowest retention value of 49% of accumulated drug was obtained for JL adria cells incubated for 15 min with drug, followed by 2 h without drug.

In similar experiments using 5, 10 or 20 µM adriamycin, each JL subline showed a relatively rapid rate of accumulation before 15 min, followed by a slower, but steady rate of accumulation up to 2 h of incubation (i.e., similar to Figure 1*c,d*). After a 1 h incubation in 5 µM adriamycin, JL control 1, control 2, AMSA and adria cells accumulated 4.5, 3.2, 4.2 and 4.1 moles/cell (× $10^{-16}$), respectively. Levels of adriamycin retention (after 30 min in drug-free medium) in drug resistant cells were not significantly different from those in control cells (data not shown). The presence of 10 mM sodium azide during either drug uptake or efflux incubation periods did not affect adriamycin accumulation or retention for JL control, AMSA or adria sublines, suggesting passive drug uptake and efflux (data not shown).

Fluorescence microscopy of JL cells incubated with 20 µM adriamycin or 5 µM daunorubicin for 1 h at 37°C revealed a predominant nuclear location of each drug in JL control, AMSA and adria cells (data not shown). Hence no significant differences in drug accumulation or retention were found between drug resistant and control cells.

### *Protein analyses*

SDS-PAGE separation of proteins sequentially extracted by NP40, DOC/Brij and SDS from $^{35}$S-met labelled JL drug-resistant and control subline showed few differences. A broad band at ~20 kDa was more diffuse in appearance in drug resistant cells, while a slightly smaller, sharp band was absent from the drug resistant cells (data not shown). Both proteins were only extracted by SDS.

Polyclonal anti-Jurkat, anti-PBLT and anti-AMSA$^R$ antisera were each used to radioimmunoprecipitate NP40-extracted proteins from Little sublines. Although several differences were apparent between sublines, none consistently correlated with drug resistance (data not shown).

NP40 extractable iodinated surface proteins revealed numerous differences between sublines (arrows in Figure 2). The only consistent resistance-associated difference was increased intensity of a protein at 85K MW, particularly from the three amsacrine resistant sublines and to a lesser extent from JL adria 200 and JB1 adria.

Neither immunoblotting (kindly performed by Dr D.R. Bell) using monoclonal antibody C219 (Kartner *et al.*, 1985) nor silver staining detected any Pgp protein from enriched membrane fractions of JL control, JL AMSA or JL adria cells.

### *Drug metabolism*

Since the glutathione detoxification system is involved in metabolism of adriamycin (Arrick & Nathan, 1984) and amsacrine (Shoemaker *et al.*, 1984), possible contribution of the system to detoxification of these drugs in resistant and control Jurkat sublines was tested. Assays of GSH content (Akerboom & Sies, 1981), and activities of GSH transferase (Warholm *et al.*, 1985), GSH peroxidase (Flohé & Gunzler,

**Figure 2** Radioiodinated proteins from drug resistant and control Jurkat sublines. Cell surface proteins were radioiodinated using the lactoperoxidase procedure. NP40 extracts of $^{125}$I-labelled cells were separated by SDS-PAGE and visualised by fluorography. Lanes were loaded with equal radioactivity. Lane 1: JL control subculture 1. Lane 2: JL control subculture 2. Lane 3: JL AMSA. Lane 4: JL adria 200. Lane 5: JL adria 500. Lane 6: JB1 B1 control. Lane 7: JB1 B1 AMSA. Lane 8: JB1 adria. Lane 9: JB2 control. Lane 10: JB2 AMSA.

**Figure 3** Time dependent production of DNR metabolites/degradation products in reaction mixtures containing $^3$H-DNR and cell homogenate. Homogenates of JL cells (4.0 mg ml$^{-1}$ protein) were incubated with 2 μM $^3$H-DNA in the presence or absence of 0.5 mM NADPH at pH 8.5 for 4.5 h or 24 h. DNR, metabolites and possible degradation products were extracted and separated by TLC. Spots were visualised by fluorography. Samples 1–8 were incubated for 4.5 h – NADPH: 1, 2 buffer only; 3, 4 Little control cell homogenate; 5, 6 JL AMSA cell homogenate; 7, 8 JL adria cells homogenate. Samples 9 to 16 were incubated for 4.5 h, + NADPH: 9, 10 buffer only; 11, 12 JL control cell homogenate; 13, 14 JL AMSA cell homogenate; 15, 16 JL adria cell homogenate. Samples 18 to 25 were incubated for 24 h, – NADPH: 18, 19 buffer only; 20, 21 JL control cell homogenate; 22, 23 JL AMSA cell homogenate; 24, 25 JL adria cell homogenate. Samples 26 to 33 were incubated for 24 h, + NADPH: 26, 27 buffer only; 28, 29 JL control cell homogenate; 30, 31 JL AMSA cell homogenate; 32,33 JL adria cell homogenate. Samples 17 and 34 contained DNR diluted into pH 8.5 buffer immediately before spotting onto the TLC plate.

1984), GSH reductase (Carlberg & Mannervik, 1985) and γ-glutamylcystein synthetase (Seelig & Meister, 1985) did not reveal any significant differences between JL control, and drug resistant sublines (data not shown). In addition, activities of other putative drug metabolising enzymes, catalase (Aebi, 1984), superoxide dismutase (Beauchamp & Fridovich, 1971), NADPH cytochrome P450 reductase (Strobel & Dignam, 1978), aryl sulphotransferase (Sekura *et al.*, 1981) and xanthine oxidase (Dodion *et al.*, 1987) were not altered in resistant compared with control JL sublines (data not shown).

An assay previously used to measure anthracycline reductase activity in human leukaemic and myelocytic cells (Ahmed, 1985) was used to compare metabolism of $^3$H-DNR by JL control and resistant sublines. Cell homogenates from JL sublines (4 mg ml$^{-1}$) were incubated at pH 8.5 with 2 μM $^3$H-DNR in the presence or absence of 0.5 mM NADPH for 4.5 h or 24 h at 37°C. Figure 3 shows representative fluorographs obtained after sample processing and TLC. The position of DNR (species I) was identified from direct application of $^3$H-DNR (samples 17 and 34). After incubation for 4.5 h, a small amount of a more slowly migrating species (species II) was present in all JL cell homogenates with added NADPH (samples 11 to 16, Figure 3). Species II was absent from all incubated mixtures containing NADPH, but no cell homogenate (samples 9, 19, 26, 27), and was also absent from all mixtures incubated without NADPH (samples 1 to 8 and 18 to 25). After a 24 h incubation, relatively more species II could be extracted from control cell homogenates (samples 28 and 29, Figure 3) than resistant cell homogenates (samples 30 to 33). For each subline, the relative amounts of species I and II extracted decreased with increasing protein concentration (data not shown), however, JL AMSA consistently contained 0.5 × and JL adria 0.3 × as much of species II as did control cells. Species II was not detected from reaction mixtures incubated in buffers between pH 5.5 and pH 8.0 for 24 h at 37°C. Chromatography of DNR and its metabolites suggested that species I was daunorubicin and species II daunorubicinol.

In similar studies of amsacrine metabolism, both resistant and control cell homogenates gave rise to identical patterns of metabolites/degradation products (pH 6.0 and 8.5, ± 0.5 mM NADPH, for up to 24 h at 37°C).

*Cytogenic analyses*

JL and JB2 control and drug resistant sublines were karyotyped by G-banding, seeking evidence for gene amplification (dmins, HSRs, or ABRs) which others have reported may accompany drug resistance. Results shown in Table II*a* indicate much karyotypic instability during selection, but little in drug resistant cells (Table II*b*). This suggests that resistant cells were also resistant to DNA damage from cytotoxic drugs. No karyotypic features were found to correlate consistently with drug resistance.

*Cytogenetic damage produced by amsacrine or adriamycin*

Table III lists the number of metaphases containing various degrees of cytogenetic damage after 1 h incubations of cultures with amsacrine or adriamycin followed by 3 h incubation in fresh medium containing colchicine. For each subline, metaphases prepared from non-drug-treated cultures contained no apparent cytogenetic damage. Drug concentrations which left approximately 50% of metaphases without damage in JL control, AMSA and adria sublines were < 1 μM, 20 μM and 50 μM amsacrine, respectively and < 1.5 μM, 5 μM and 25 μM adriamycin, respectively. Pulverisation of some chromosomes occurred after treatment of control cells with amsacrine or adriamycin, and after treatment of JL AMSA cells with amsacrine but not adriamycin. Generally, drug treatment did not cause pulverisation of JL adria meta-

**Table II** Numbers of chromosomes and dmins in resistant, partially resistant, and control Jurkat sublines

| *Subline* | | *[Drug]nM* | *Weeks at this [drug]* | *No. of spreads* | *Chromosomes /cell-range* | *Chromosomes /cell-mean* | *Chromosomes /cell-SD* | *Metaphases with dmins* | *Range dmins/cell* |
|---|---|---|---|---|---|---|---|---|---|
| **a** JB2 | con | – | – | 34 | 67–250 | 90 | 29 | 2 | 1,2 |
| | AMSA | 200 | 4 | 25 | 50–108 | 81 | 10 | 14 | 1–4 |
| | adria | 50 | 4 | 16 | 67–135 | 86 | 15 | 8 | 1,2,13 |
| JL | con | – | – | 21 | 46–95 | 50 | 11 | 0 | – |
| | AMSA | 200 | 8 | 24 | 45–93 | 53 | 14 | 2 | 1,2 |
| | adria | 50 | 4 | 19 | 45–112 | 60 | 20 | 8 | 1–7 |
| JL | con1 | – | – | 20 | 44–131 | 55 | 22 | 0 | – |
| | con2 | – | – | 23 | 48–132 | 86 | 18 | 1 | 1 |
| | AMSA | 1000 | 6 | 29 | 42–205 | 67 | 48 | 3 | 1–5 |
| | adria | 200 | 3 | 19 | 40–187 | 82 | 52 | 5 | 2–5 |
| **b** JL | con1 | – | – | 11 | 44–91 | 51 | 24 | 2 | 1 |
| | con2 | – | – | 10 | 45–93 | 77 | 22 | 0 | – |
| | AMSA | 1000 | 42 | 11 | 42–46 | 45 | 1.1 | 2 | 1 |
| | adria | 200 | 42 | 10 | 43–46 | 45 | 1.1 | 0 | – |

Section a of the table includes data from partially resistant sublines and section b data from resistant sublines. In each case metaphase spreads of control sublines were analysed at the same time as the corresponding resistant/partially resistant sublines.

**Table III** Resistance to DNA damage – cytogenetic assay

| *Subline* | *[Drug]* | *Metaphases scored* | *% With no damage* | *% With 1 chromatid break* | *% With 2 chromatid breaks* | *% With 3/+ chromatid breaks* | *% With chromatid exchange* | *% With chromatid exchange + breaks* | *% With dmins only* | *% Pulverised* |
|---|---|---|---|---|---|---|---|---|---|---|
| JL control | 1 μM AMSA | 139 | 36 | 7 | 7 | 16 | 7 | 12 | 7 | 8 |
| | 2 μM AMSA | 20 | 10 | 15 | 25 | 15 | 0 | 15 | 5 | 15 |
| | 1.5 μM adria | 90 | 39 | 13 | 4 | 30 | 0 | 2 | 2 | 10 |
| | 2 μM adria | 51 | 35 | 8 | 8 | 33 | 0 | 2 | 2 | 12 |
| JL AMSA | 20 μM AMSA | 164 | 47 | 12 | 05 | 18 | 1 | 2 | 4 | 11 |
| | 30 μM AMSA | 91 | 35 | 16 | 13 | 15 | 1 | 2 | 13 | 5 |
| | 5 μM adria | 80 | 53 | 5 | 24 | 17 | 0 | 0 | 1 | 0 |
| | 10 μM adria | 70 | 36 | 17 | 13 | 34 | 0 | 0 | 0 | 0 |
| | 15 μM adria | 40 | 20 | 15 | 20 | 45 | 0 | 0 | 0 | 0 |
| JL adria | 40 μM AMSA | 41 | 71 | 12 | 0 | 7 | 0 | 5 | 2.5 | 2.5 |
| | 50 μM AMSA | 40 | 45 | 25 | 15 | 10 | 2.5 | 2.5 | 0 | 0 |
| | 10 μM adria | 50 | 64 | 18 | 14 | 8 | 2 | 0 | 4 | 0 |
| | 20 μM adria | 140 | 56 | 18 | 10 | 8 | 1 | 1 | 6 | 0 |
| | 25 μM adria | 40 | 50 | 22.5 | 17.5 | 10 | 0 | 0 | 0 | 0 |

JL control, JL AMSA and JL adria cultures were treated for 1 h at 37°C with various concentrations of amsacrine or adriamycin then resuspended in fresh medium containing colchicine. After a further 3 h incubation at 37°C, metaphase spreads were prepared and scored for cytogenetic damage. Data were derived from numerous experiments.

**Table IV** Effect of araC on resistance to DNA damage – cytogenetic assay

| *Subline* | *Treatment before araC* | *Metaphases scored* | *% With no damage* | *% With 1 chromatid break* | *% With 2 chromatid breaks* | *% With 3/+ chromatid breaks* | *% With chromatid exchange* | *% With chromatid exchange + breaks* | *% With dmins only* | *% Pulverised* |
|---|---|---|---|---|---|---|---|---|---|---|
| JL control | – | 30 | 93 | 0 | 0 | 0 | 0 | 7 | 0 | 0 |
| | 1 μM AMSA | 44 | 9 | 7 | 5 | 34 | 0 | 9 | 4 | 32 |
| | 2 μM adria | 20 | 0 | 0 | 0 | 35 | 0 | 0 | 0 | 65 |
| JL AMSA | – | 62 | 81 | 11 | 1.5 | 1.5 | 0 | 0 | 5 | 0 |
| | 20 μM AMSA | 73 | 23 | 22 | 8 | 27 | 2 | 2 | 5 | 11 |
| JL adria | – | 35 | 77 | 11 | 9 | 3 | 0 | 0 | 0 | 0 |
| | 10 μM adria | 20 | 40 | 0 | 15 | 40 | 0 | 0 | 5 | 0 |
| | 20 μM adria | 30 | 43 | 20 | 10 | 20 | 0 | 0 | 7 | 0 |

JL control, JL ASMA and JL adria cultures were treated for 1 h at 37°C with various concentrations of amsacrine or adriamycin then resuspended in fresh medium containing colchicine and 50 μM ara C. After a further 3 h incubation at 37°C, metaphase spreads were prepared and scored for cytogenetic damage.

**Table V** Effect of novobiocin on resistance to DNA damage – cytogenetic assay

| *Subline* | *Treatment following novobiocin* | *Metaphases scored* | *% With no damage* | *% With 1 chromatid break* | *% With 2 chromatid breaks* | *% With 3/+ chromatid breaks* | *% With chromatid exchange* | *% With chromatid exchange + breaks* | *% With dmins only* | *% Pulverised* |
|---|---|---|---|---|---|---|---|---|---|---|
| JL control | – | 101 | 79 | 11 | 3 | 1 | 0 | 0 | 6 | 0 |
| | 1μM AMSA | 60 | 53 | 17 | 7 | 8 | 3 | 5 | 5 | 2 |
| | 1.5μM adria | 25 | 40 | 20 | 12 | 20 | 0 | 0 | 8 | 0 |
| JL AMSA | – | 40 | 90 | 7.5 | 2.5 | 0 | 0 | 0 | 0 | 0 |
| | 20μM AMSA | 40 | 50 | 22.5 | 0 | 15 | 0 | 2.5 | 2.5 | 7.5 |
| | 30μM AMSA | 30 | 40 | 23 | 0 | 13 | 4 | 0 | 0 | 0 |
| JL adria | – | 40 | 80 | 17.5 | 0 | 2.5 | 0 | 0 | 0 | 0 |
| | 20μM adria | 31 | 52 | 26 | 3 | 13 | 0 | 0 | 6 | 0 |
| | 25μM adria | 42 | 67 | 14 | 7 | 10 | 0 | 2 | 0 | 0 |

JL control, JL AMSA and JL adria sublines were treated for 30 min with 1 mM novobiocin at 37°C, centrifuged, resuspended in medium containing no drug, amsacrine or adriamycin for 1 h, then resuspended in medium containing colchicine. After a further 3 h, metaphase spreads were prepared and scored for cytogenetic damage.

phases, even at very high drug concentrations which reduced the number of metaphases to less than 10% of the number from parallel untreated cultures.

AraC has previously been shown to inhibit DNA repair of chromosome aberrations (Preston, 1982), and it was used here as one means of ascertaining if DNA repair was increased in drug resistant cells. Comparing Tables III and IV, and considering cytogenetic damage caused by araC alone, it is clear that araC augmented damage produced by adriamycin or amsacrine treatment of JL control cells. Although post-treatment with araC also decreased the fraction of undamaged JL AMSA metaphases seen after amsacrine treatment, the effect was smaller than for control cells. AraC post-treatment did not significantly affect the amount of damage caused by incubation of JL adria cells with adriamycin (when damage induced by ara C alone was considered).

Novobiocin inhibits binding of ATP to topoisomerase II, thus inhibiting strand-passage activity and topoisomerase turnover which require ATP binding and hydrolysis (Vosberg, 1985). It also inhibits several other ATP dependent enzymes. Previous experiments showed the growth of JL control, JL AMSA and JL adria sublines were similarly retarded by novobiocin. Each subline showed an $IC_{50}$ of approximately 200 μM novobiocin in 3-day continuous exposure experiments (data not shown).

Table V shows the cytogenetic damage produced when cultures were treated with 1 mM novobiocin just prior to drug exposure. Pretreatment of JL control cells with novobiocin reduced the level of cytogenetic damage induced by amsacrine or adriamycin but had no effect in preventing damage to drug resistant cells. (Statistical analysis of results in Tables III–V indicates that these conclusions are statistically highly significant).

*Quantification of DNA breaks using a fluorescence assay*

In this assay, F values represent the fraction of duplex DNA versus total DNA remaining after treatment of cells with alkali to cause unwinding of DNA at break sites. Figure 4 shows the F values obtained after treatment of JL control, AMSA, and adria with various concentrations of amsacrine for 1 h. F values for untreated cells were probably less than one because of inherent nicks in cellular DNA and a small percentage of dead cells in the culture used. Amsacrine concentrations required to reduce F values to 80% of the non-drug-treated values were 0.12 μM, 11 μM and 7.5 μM for JL control, JL AMSA and JL adria sublines, respectively.

Figure 4 also shows F values obtained after treatment of JL control (Figure 4a,) JL AMSA (Figure 4b) and JL adria (Figure 4c) sublines with various concentrations of adriamycin for 1 h. From these curves, the adriamycin concentrations required to reduce F values to 80% of the non-drug-treated values were 4.5 μM, 19 μM and 16 μM for JL control, JL AMSA and JL adria sublines, respectively.

Damage to JL AMSA or JL adria DNA caused by 10 μM and 7 μM amsacrine, respectively, was not significantly repaired after 2 h in drug-free medium (Figure 5a). In contrast, damage to JL control DNA (in cells incubated with 0.2 μM amsacrine) was totally repaired within 1.5 h of incubation in drug-free medium. In all sublines treated with adriamycin, production of DNA breaks continued for up to 30 min after removal of extracellular drug (Figure 5b). After this time, each of the sublines showed only slight DNA repair.

*Drug-induced PDC formation*

Figure 6 shows stimulation of PDC formation in JL cells treated with amsacrine for 1 h at 37°C. In this experiment, 2.5 fold stimulation of PDC formation required 0.7 μM, 43 μM and 5 μM amsacrine using JL control, JL AMSA and JL adria sublines, respectively.

Adriamycin had an unexpected effect on PDC formation. For each subline, stimulation of PDC formation was less after the 2 h incubation (Figure 7b) than the 1 h incubation (Figure 7a). Furthermore, for the drug resistant sublines, fold-stimulation was less than 1, suggesting that adriamycin treatment caused more DNA to pass through the filters. Maximum stimulation of PDC formation in control cells after a 1 h adriamycin treatment was approximately 2.3-fold (Figure 7a), compared with approximately 18-fold stimulation caused by a 1 h amsacrine treatment (Figure 6).

*Drug-DNA binding within resistant and control cells*

Although drug accumulation and retention were unaltered in resistant compared with control cells, it seemed possible that reduced association of the intercalating drugs with DNA could affect resistance of JL AMSA and JL adria cells to DNA damaging effects.

Hoechst-33342 fluorescence of cell suspensions or calf thymus DNA solutions is stable for several hours at 37°C. Uptake and retention of amsacrine or adriamycin were unaffected by preincubation of cells with Hoechst 33342 (data not shown).

Figure 8 shows Hoechst fluorescence remaining after post-incubation with various concentrations of amsacrine for 1 h. Amsacrine concentrations required to reduce Hoechst fluorescence to 50% of the non-amsacrine-treated values were 28, 25, 23, 24 and 19 μM for JL control 1, control 2, AMSA, adria sublines and DNA, respectively, indicating similar amsacrine-DNA binding in each case. Quenching of Hoechst fluorescence in a DNA solution was only slightly more efficient than quenching within cells. Maximum quenching of Hoechst fluorescence occurred within 10 min of adding amsacrine to cells or DNA. Approximately 85% of the initial fluorescence was restored within 5 min of transferring cells to amsacrine-free medium, and fluorescence changed little during the next hour. Although Hoechst fluorescence recovery was high, 75% of the amsacrine accumulated during the 1 h drug incubation remained in the cells. This retained amsacrine may represent drug sequestered into an extranuclear compartment as previously described by Zwelling *et al.* (1982).

**Figure 4** Fluorescence assay for DNA damage in the cells treated with amsacrine —□— or adriamycin —◆—. **a**, JL control cells; **b**, JL AMSA cells; **c**, JL adria cells. Cells were incubated with various concentrations of drug in PBS/5% FCS at 37°C for 1 h. DNA breakage was then determined using a fluorescence assay. Each point is the mean of four experiments, and standard deviations did not exceed ± 9%. Decreasing fluorescence (F) values represent increasing DNA breakage. Note that the relationship between DNA breakage and fluorescence is only linear when data is plotted on a log/log basis.

**Figure 5** Repair of DNA damage in JL cells previously treated with amsacrine or adriamycin. JL control (▲), JL AMSA (■) and JL adria (□) cells were treated for 1 h with 0.2 μM, 10 μM and 7 μM amsacrine, respectively **a**, or 6 μM, 18 μM and 18 μM adriamycin, respectively **b**. After various post-incubation periods in drug-free PBS/5% FCS, the fluorescence assay was used to assess DNA breakage. The repair ratio was calculated from:

$$\frac{\text{F value of cells + drug}}{\text{F value of cells-drug}}$$

using cells incubated for identical lengths of time in PBS/5% FCS. For each drug treatment, the mean repair ratio was calculated from two independent experiments, each containing duplicate incubations with each concentration of drug.

Hoechst fluorescence was reduced to 50% of the non-adriamycin-treated values by a 1 h exposure to 1.9, 7.2, 3.2, 4.3 and 1.4 μM adriamycin for JL control 1, control 2, AMSA and adria cells and DNA, respectively (data not shown). Since resistant values fell between control values, it appears that drug binding did not differ significantly between drug resistant and control cells. In contrast to quenching by amsacrine, adriamycin-induced quenching was irreversible (after reincubation of cells in drug-free medium for 30 min).

*Comparisons between DNA damaging and cytotoxic effects of drugs*

It was noted that resistance to cytogenetic damage by drugs did not reflect the resistance of JL adria and JL AMSA sublines to drug cytotoxicity during 3 day continuous exposures. Thus, the JL AMSA subline appeared less resistant to adriamycin- or amsacrine-induced cytogenetic damage than the Little adria subline, whereas the JL AMSA subline was more resistant to amsacrine than the JL adria subline in the cytotoxicity assay. Because of this discrepancy, growth inhibitory effects of amsacrine and adriamycin were also measured after 1 h incubations of cultures with drug at 37°C, followed by centrifugation and resuspension in drug-free medium for 3 days. Table VI shows drug cytotoxicities for 3 day and 1 h exposures and also summarises DNA damaging effects from preceding sections (to facilitate comparisons). Generally, R factors after 1 h drug exposure were less than

**Figure 6** Stimulation of PDC formation in JL cells treated with amsacrine. $^3$H-thymidine labelled JL control —⊡—, AMSA —◆— and adria —■— cells were incubated with various concentrations of amsacrine in RPMI medium for 1 h at 37°C, then used in an assay for PDC formation. Fold-stimulation of PDCs was calculated from the ratio:

$$\frac{^3\text{H bound to filters using drug treated cells}}{^3\text{H bound to filters using untreated cells}}$$

Standard deviations were calculated from triplicate samples.

**Figure 7** Stimulation of PDC formation in JL cells treated with adriamycin. $^3$H-thymidine labelled JL control (▲), JL AMSA (■) and JL adria (□) cells were incubated with various concentrations of adriamycin in RPMI medium for 1 h **a**, or 2 h **b**, at 37°C. Using the assay for PDC formation, fold-stimulation of PDCs was calculated from:

$$\frac{^3\text{H bound to filters using drug treated cells}}{^3\text{H bound to filters using untreated cells}}$$

Standard deviations were calculated from triplicate samples.

**Figure 8** Quenching of Hoechst-33342 fluorescence by amsacrine. Suspensions of cells ($10^6$ cells/ml) from JL control 1 (▲), control 2 (○), AMSA (□) and adria (■) sublines and a solution of calf thymus DNA ($10\,\mu g\,ml^{-1}$) (△) were incubated with Hoechst-33342 for 1 h then incubated with various concentrations of amsacrine for 1 h. Hoechst fluorescence for each sample was then measured. Each point represent the mean of duplicate samples. (Standard deviations did not exceed ± 8%).

those from 3 day continuous exposure cytotoxicity experiments. Most notably, resistance of the JL AMSA subline to adriamycin cytotoxicity was negligible in the 1 h exposure assay. Also, the 1 h cytotoxicity assay did not reflect the relatively greater resistance of the JL adria subline to amsacrine-induced cytogenetic damage compared with the JL AMSA subline.

Since production of $H_2O_2$ during adriamycin metabolism may cause DNA breakage (Goormaghtigh & Ruysschaert, 1984), cytotoxicity of $H_2O_2$ to resistant and control sublines was assayed in the 3 day cell growth assay. Average R factors were 1.4 for the JL AMSA subline and 1.3 for the JL adria subline. The fluorescence assay was used to measure DNA breakage caused by incubation of JL sublines with various concentrations of $H_2O_2$ for 1 h at 37°C. Concentrations of $H_2O_2$ required to reduce the F values to 80% of the values for untreated cells were 60 μM, 110μM and 120 μM for JL control, JL AMSA and JL adria sublines, respectively. Assays for $H_2O_2$ induced cytogenetic damage were unsuccessful, in that $H_2O_2$ reduced metaphases without causing aberrations in all three cell lines.

### Discussion

Although development of resistance to adriamycin or amsacrine was accompanied by cross resistance to several other drugs, the resistant Jurkat sublines characterised in detail here did not display the MDR phenotype previously described by others (Beck, 1987; Moscow & Cowan, 1988; Bradley *et al.*, 1988). Thus, comparing resistant Jurkat sublines to control drug sensitive sublines showed that drug transport was unaltered, Pgp was not overexpressed, neither a calcium channel blocker nor calmodulin inhibitor reversed resistance and there was no evidence (in the form of HSRs or ABRs) for gene amplification at the Pgp gene locus on the q arm of chromosome 7. Despite these findings, it was noted that drug resistance levels were similar to those of cell lines previously shown to be MDR (Dalton *et al.*, 1986). One line we have not yet studied in any detail, JB1 adria, may exhibit MDR with Pgp overexpression since it is cross resistant to vincristine.

Although there was no significant difference between JL resistant and control sublines in amsacrine (or adriamycin) accumulation or retention, Figure 1 does show interesting kinetics of amsacrine accumulation. At lower concentrations of drug (Figure 1), intracellular amsacrine peaked then decreased whereas at higher drug concentrations intracellular amsacrine did not reach plateau levels during the incubation time and intracellular binding sites were not saturated.

For each of the four JL sublines incubated with 1 μM to 10 μM amsacrine for 1 h, intracellular to extracellular drug concentration ratios increased from an average of 162 ± 8 to 343 ± 13. Therefore, Jurkat cells exhibited cooperative sequestration of amsacrine as previously described for L1210 cells incubated with this drug (Zwelling *et al.*, 1982), and our results are in broad agreement with their postulated model of drug movement. The site of drug sequestration cannot be determined from these experiments but the Hoechst quenching reversal results reinforce Zwelling's suggestion that drug is sequestered apart from DNA. JL control or resistant sublines did not show co-operative sequestration of adriamycin since intracellular to extra-cellular drug concentration ratios steadily decreased with increasing extracellular adriamycin concentrations (between 5 and 20 μM).

Comparisons of proteins revealed two protein differences between sublines which consistently correlated with the presence of drug resistance. Firstly, there were changes among small polypeptides ($<$20K MW) extracted by SDS. Van der Bliek *et al.* (1986) has detected elevated expression of the 19–20K MW calcium-binding cytosolic protein sorcin in resistant cells. However, since differences between Jurkat sublines were not detected in non-ionic detergent extracts, it is unlikely that differences in our cells involved sorcin.

The second resistance-associated difference was an increased amount of an 85K MW protein from JL AMSA, JB1 AMSA, JB2 AMSA, JL adria 200 and JB1 adria compared with drug-sensitive sublines, seen only after NP40 extraction of radioiodinated surface proteins (Figure 2). Recently, Hamada *et al.* (1988) described overexpression of an 85K MW membrane protein in two adriamycin resistant human tumour cell lines. Antibodies to this protein specifically inhibited growth of resistant cells but did not affect drug accumulation, so the functional role of the 85K MW protein was not clear. It would be interesting to determine if our 85K MW protein is related to that observed by Hamada *et al.*

From a large range of protein comparisons between resistant and control Jurkat sublines (most data not shown here), numerous protein differences occurred which were not consistently associated with drug resistance, but were expressions of phenotypic drift. Hence, it was essential to compare more than one set of control and resistant sublines in deciding which changes were drug resistance-related. A similar comment applies to possible cytogenetic correlates of drug resistance. For instance JL AMSA possessed rep(5;13), rep(7;13) and 2der(3) chromosomes. Comparing this line alone with JL control would have made these translocations appear significant in resistance. However, the other drug resistant lines studies including a subline of JL AMSA named JL AM, showed other aberrations.

Numerous assays for enzyme activities suggested that elevated GSH-related detoxification or altered cytochrome P450 functions did not contribute towards drug resistance of Jurkat sublines. In contrast, MDR MCF 7 human breast cancer cells (selected for doxorubicin resistance) contained elevated GSH transferase, GSH peroxidase, UDP glucuronyltransferase and sulpho-transferase activities, and reduced cytochrome P450 inducibility compared with wild type cells (Cowan *et al.*, 1986; Sinha *et al.*, 1987). Resistant JL cell homogenates contained less of an NADPH-dependent activity which produced a more slowly migrating species (probably daunorubicinol) from $^3$H-DNR (Figure 3). The pH optimum (close to pH 8.5) and NADPH dependence of the activity are consistent with the presence of DNR reductase activity (Ahmed, 1985). Ahmed suggested that intracellular conversion of daunorubicin to daunorubicinol may favour cell killing since lower lipophilicity of the cytotoxic 13-dihydro derivatives would favour intracellular drug retention. Indeed, in a study of daunorubicin resistant human myelocytic cells (Vasanthakumar & Ahmed, 1986), it was suggested that decreased DNR reductase activity could contribute towards drug resistance. Thus, selection for adriamycin resistant JL cells may have selected for cells containing less aldo-keto reductase activity. However, it is unclear how selec-

**Table VI** Collated data

| *Drug* | | *Amsacrine* | | | *Adriamycin* | |
|---|---|---|---|---|---|---|
| *Assay (units)* | *JL control* | *JL AMSA (R factor)* | *JL adria (R factor)* | *JL control* | *JL AMSA (R factor)* | *JL adria (R factor)* |
| 3 day cytotoxicity $IC_{50}$ (μM) | 0.078 | 3.6 (47) | 2.0 (28) | 0.3 | 0.146 (5) | 0.329 (11) |
| 1 h cytotoxicity $IC_{50}$ (μM) | 1.6 | 40 (25) | 30 (18) | 1.8 | 1.6 (0.9) | 6 (4.5) |
| 1 h cytogenetic damage: 50% undamaged chromosomes (μM) | <1 | 20 (>20) | 50 (>50) | <1.5 | 5 (>3.3) | 25 (>17) |
| 1 h DNA breakage (FADU): 80% duplex DNA (μM) | 0.12 | 11 (92) | 7.5 (63) | 4.5 | 19 (4.2) | 16 (3.5) |
| PDC formation -2.5 fold stim (μM) | 0.7 | 43 (61) | 5 (7.1) | – | – | – |
| -5 fold stim (μM) | 1.3 | >50 (>38) | 20 (15.4) | – | – | – |
| -plateau stim level (×) | 18 | 2.8 (6.4) | 7.5 (2.4) | – | – | – |

Summary of cytotoxic and DNA damaging effects of amsacrine and adriamycin with JL sublines. Cytotoxicity and DNA damaging effects of drugs were assayed and compared using the criteria shown in the far left column. R factors of resistant sublines were calculated by dividing the drug concentration required to cause a certain level of cytotoxicity or DNA damage to resistant cells by the concentration required to cause an identical effect with control cells.

tion for amsacrine resistance would select cells with altered anthracycline metabolism. Possibly, the NADPH dependent difference between resistant and control cells is mediated by a factor other than daunorubicin reductase.

A fluorescence assay for DNA damage, a cytogenetic aberration assay and an assay for DNA-protein complexes all showed clearly that drug resistant cells were resistant to DNA damage induced by either amsacrine or adriamycin. Inhibiting DNA repair with araC showed that repair occurred in control cells but not resistant cells. Likewise, the fluorescence assay for DNA damage (Figure 5) indicated that control cells repaired more amsacrine induced damage than did resistant cells. A caveat in both sets of results is the possibility that high concentrations of drugs needed to produce effects in drug resistant cells may inhibit the DNA repair processes in those cells. An additional possibility is that DNA repair played a role in cell death. However, we have no evidence that enhanced repair played a role in drug resistance.

Although novobiocin is not a very specific inhibitor of topo II, its use prior to cytotoxic drug exposure did affect cells differentially, in that it reduced chromosome damage in control cells but not in drug resistant cells.

Amsacrine and adriamycin resistant sublines were slightly (less than two-fold) resistant to $H_2O_2$ induced DNA breakage compared with control cells. The mechanism of this effect is unclear, since levels of protecting enzymes are not elevated in resistant cells. Although adriamycin has long been recognised as having a free radical component in its mode of action, no significant resistance to this mode of action is apparent in any of the cells studied here.

Quenching by cytotoxic drugs of DNA stained with fluorescent dye Hoechst 33342 revealed no significant differences between drug resistant, control cells, and DNA in solution. These results confirm that drug resistance is not mediated by altered drug accumulation, transport, or reduced accessibility to DNA in resistant cells. Removing cells from amsacrine resulted in rapid restoration of Hoechst fluorescence, but only limited efflux of amsacrine from cells. Hence, most of the amsacrine is sequestered at unknown sites in the cells, presumably at quite a high concentration. In contrast, adriamycin remained bound to DNA after removing cells from drug solutions. Presumably, this tighter binding underlies the ability of adriamycin to keep on causing breaks (Figure 5b), and the apparent absence of repair seen here as well as by others (Zwelling *et al.*, 1981; Robson *et al.*, 1987).

Both drug resistant JL sublines were significantly resistant to stimulation of protein associated DNA breaks by amsacrine compared with the control subline (Figure 6). The JL AMSA subline was more resistant than the JL adria subline, reflecting relative resistances of these sublines to cytotoxic effects of amsacrine. Thus, although the relationship between cytotoxicity and PDC formation is unclear, resistance of JL AMSA and JL adria sublines to amsacrine could at least partially be mediated by resistance to PDC formation. Since amsacrine produces PDCs by interaction with topoisomerase II (Zwelling, 1985), it seems likely that the drug-topoisomerase-DNA interaction is altered in drug resistant JL sublines. While it is not surprising that drug resistant JL sublines resisted stimulation of PDC formation by adriamycin (Figure 7a), it was unexpected that adriamycin treated resistant cells apparently contained fewer PDCs than untreated cells. It was also unusual that a 2 h incubation of resistant or control JL cells with adriamycin apparently gave less stimulation of PDC production than a 1 h incubation (Figure 7). One explanation is that progressive fragmentation of DNA involving non-protein-associated breaks (perhaps involving free radicals) occurred during the adriamycin treatment. Thus, greater fragmentation of DNA would reduce the amount of radiolabelled DNA in PDC complexes on filters, such that PDC formation seemed lower.

Although in general the results were consistent with the hypothesis that drug resistance is effected by alterations in DNA-topoisomerase II-drug interactions, the data also suggest differences in the mode of action, or resistance to amsacrine and adriamycin between JL AMSA and JL adria.

In all assays, JL adria cells were more resistant to amsacrine than to adriamycin although they were selected for resistance to adriamycin. JL AMSA cells, were more resistant to amsacrine than adriamycin in all assays (Table VI). Thus, correlations with drugs rather than resistant cell type are observed. This data strongly suggests that adriamycin acts in part by a mechanism that is shared with amsacrine and partly by a distinct mechanism, to which the adriamycin resistant cells remain largely susceptible. Developing resistance to adriamycin was more difficult than to amsacrine. The peculiarities of PDC formation with adriamycin, and the likelihood that the drug causes breaks in DNA apart from those induced by topoisomerase II are consistent with this possibility. Differences in amsacrine and adriamycin binding to DNA as shown by the Hoechst fluorescence quenching experiments have also been outlined.

In addition, Table VI contains several quite striking anomalies. For instance, in the fluorescence assay for DNA damage, JL AMSA was more resistant to adriamycin than JL adria, although JL adria was more resistant to adriamycin than JL AMSA in all other assays. Comparing the relative concentrations of amsacrine and adriamycin required to produce similar levels of cytotoxicity or DNA damage to the JL control subline (Table VI): in cytotoxic or cytogenetic damage assays, concentrations of amsacrine and adriamycin required to produce similar effects were less than 3-fold different, but amsacrine was approximately 40-fold more potent than adriamycin in producing DNA damage detected by the fluorescence assay. Resistance of JL adria to cytogenetic damage by amsacrine was greater than that of JL AMSA, although JL AMSA was more resistant to amsacrine in all other assays. This resistance was not associated with commensurate resistance to PDC formation, so the effect did not involve topoisomerase II. Since the cells had little resistance to $H_2O_2$, resistance to free radical action was not involved either. One possibility is that amsacrine and adriamycin have other modes of action, not associated with topoisomerase II or free radical action, to which the cells have some resistance. For instance, amsacrine present at high concentration in the sequestration site could affect other cellular processes. The present results suggesting some differences in the details of resistance between JL AMSA and JL adria cells have been reinforced by our recent findings of differences in their patterns of resistance to amsacrine analogues (Finlay *et al.*, 1990).

The authors wish to thank Dr D.R. Bell for performing C219 immunoblotting and also the Cancer Research Laboratory, University of Auckland School of Medicine, and Farmitalia Carlo Erba Ltd for providing samples of cytotoxic drugs. We gratefully acknowledge the financial support of the Cancer Society of New Zealand (Inc) and the Auckland University Research Committee, and thank Jean Parrott for typing the manuscript.

### References

AEBI, H. (1984). Catalase *in vitro*. *Methods Enzymol*, **105**, 121.

AHMED, N.K. (1985). Daunorubicin reductase activity in human normal lymphocytes, myeloblasts and leukemic cell lines. *Eur. J. Cancer Clin. Oncol.*, **21**, 1209.

AHMED, N.K., FELSTED, R.L. & BACHUR, N.R. (1978). Heterogeneity of anthracycline antibiotic carbonyl reductase in mammalian livers. *Biochem. Pharmacol.*, **27**, 2713.

AKERBOOM, T.P.M. & SIES, H. (1981). Assay of glutathione, glutathione disulfide, and glutathione mixed disulfides in biological samples. *Methods Enzymol.*, **77**, 373.

AMES, G.F.-L. (1986). The basis of multidrug resistance in mammalian cells: homology with bacterial transport. *Cell*, **47**, 323.

ANDERSSON, B.S., BERAN, M., BARLOGIE, B., VAN, N.T. & MCCREDIE, K.B. (1986). Analysis of nuclear *m*AMSA content by DNA fluorochrome competition. *Eur. J. Cancer Clin. Oncol.*, **22**, 883.

ARRICK, B.A. & NATHAN, C.F. (1984). Glutathione metabolism as a determinant of therapeutic efficacy: a review. *Cancer Res.*, **44**, 4224.

BEAUCHAMP, C. & FRIDOVICH, I. (1971). Superoxide dismutase: improved assays and an assay applicable to polyacrylamide gels. *Anal. Biochem.*, **44**, 276.

BECK, W.T. (1987). The cell biology of multiple drug resistance. *Biochem. Pharmacol.*, **36**, 2879.

BECK, W.T., CIRTAIN, M.C., DANKS, M.K. & 4 others (1987). Pharmacological, molecular and cytogenetic analysis of 'atypical' multidrug resistant human leukemic cells. *Cancer Res.*, **47**, 5455.

BRADFORD, M.M. (1976). A rapid and sensitive method for the quantitation of microgram quantities of protein utilizing the principle of protein-dye binding. *Anal. Biochem.*, **72**, 248.

BRADLEY, G., JURANKA, P.F. & LING, V. (1988). Mechanism of multidrug resistance. *Biochim. Biophys. Acta.*, **948**, 87.

CARLBERG, I. & MANNERVIK, B. (1985). Glutathione reductase. *Methods Enzymol.*, **113**, 484.

COWAN, K.H., BATIST, G., TULPULE, A., SINHA, B.K. & MYERS, C.E. (1986). Similar biochemical changes associated with multidrug resistance in human breast cancer cells and carcinogen-induced resistance to xenobiotics in rats. *Proc. Natl. Acad. Sci. USA*, **83**, 9328.

CURT, G.A., CLENDENIUM, N.J. & CHABNER, B.A. (1984). Drug resistance in cancer. *Cancer Treat. Rep.*, **68**, 87.

DALTON, W.S., DURIE, B.G.M., ALBERTS, D.S., GERLACH, J.H. & CRESS, A.E. (1986). Characterization of a new drug-resistant human myeloma cell line that expresses P-glycoprotein. *Cancer Res.*, **46**, 5125.

DANKS, M.I.K., YALOWICH, J.C. & BECK, W.T. (1987). Atypical multiple drug resistance in a human leukemic cell line selected for resistance to teniposide (VM-26). *Cancer Res.*, **47**, 1297.

DODION, P., BERNSTEIN, A.L., FOX, B.M. & BACHUR, N.R. (1987). Loss of fluorescence by anthracycline antibiotics: effects of xanthine oxidase and identification of the non-fluorescent metabolites. *Cancer Res.*, **47**, 1036.

FAIRCHILD, C.R., IVY, S.P., KAO-SHAN, C.-S. & 6 others (1987). Isolation of amplified and overexpressed DNA sequences from adriamycin-resistant human breast cancer cells. *Cancer Res.*, **47**, 5141.

FINLAY, G.J., BAGULEY, B.C., SNOW, K. & JUDD, W. (1990). Multiple patterns of resistance of human leukaemic cell sublines to amsacrine analogues. *J.N.C.I.*, **82**, 662.

FLOHÉ, L. & GUNZLER, W.A. (1984). Assays of glutathione peroxidase. *Methods Enzymol.*, **105**, 114.

GOORMAGHTIGH, E. & RUYSSCHAERT, J.M. (1984). Anthracycline glycoside-membrane interactions. *Biochim. Biophysic. Acta*, **779**, 271.

HAMADA, H., OKOCHI, E., WATANABE, M. & 4 others (1988). $M_R$ 85,000 membrane protein specifically expressed in adriamycin-resistant human tumour cells. *Cancer Res.*, **48**, 7082.

ISCN; AN INTERNATIONAL SYSTEM FOR HUMAN CYTOGENETIC NOMENCLATURE (1978). Defined in *Cytogen. Cell Genet.*, **21**, 309.

KANTER, P.M. & SCHWARTZ, H.S. (1982). A fluorescence enhancement assay for cellular DNA damage. *Mol. Pharmacol.*, **22**, 145.

KARTNER, N., EVERNDEN-PORELLE, D., BRADLEY, G. & LING, V. (1985). Detection of P-glycoprotein in multidrug-resistant cell lines by monoclonal antibodies. *Nature*, **316**, 820.

KEES, U.R. (1987). Resistance to 1-$\beta$-D-arabinofuranosylcytosine after high-dose treatment in childhood lymphoblastic leukemia: isolation of a drug resistant and a sensitive cell line. *Cancer Res.*, **47**, 3088.

KESSEL, D. & WHEELER, C. (1984). mAMSA as a probe for transport phenomena associated with anthracycline resistance. *Biochem. Pharmacol.*, **33**, 991.

KRISHAN, A., SAUERTEIG, A. & GORDON, K. (1985). Effect of amphotericin B on adriamycin transport in P388 cells. *Cancer Res.*, **45**, 4097.

MERRY, S., FETHERSTON, C.A., KAYE, S.B., FRESHNEG, R.I. & PLUMB, J.A. (1986). Resistance of human glioma to adriamycin *in vitro*: the role of membrane transport and its circumvention with verapamil. *Br. J. Cancer*, **53**, 129.

MOSCOW, J.A. & COWAN, K.H. (1988). Multidrug resistance. *J. Natl Cancer Inst.*, **80**, 14.

PER, S.R., MATTERN, M.R., MIRABELLI, C.K., DRAKE, F.H., JOHNSON, R.K. & CROOKE, S.T. (1987). Characterization of a subline of P388 leukemia resistant to amsacrine: evidence of altered topoisomerase II function. *Mol. Pharmacol.*, **32**, 17.

POMMIER, Y., KERRIGAN, D., SCHWARTZ, R.E., SWACK, J.A. & MCCURDY, A. (1986). Altered DNA topoisomerase II activity in Chinese hamster cells resistant to topoisomerase II inhibitors. *Cancer Res.*, **46**, 3075.

PRESTON, R.J. (1982). The use of inhibitors of DNA repair in the study of anti-tumor agents. *Cytogenet. Cell Genet.*, **33**, 20.

ROBSON, C.N., HOBAN, P.R., HARRIS, A.L. & HICKSON, I.D. (1987). Cross-sensitivity to topoisomerase II inhibitors in cytotoxic drug-hypersensitive Chinese hamster ovary cell lines. *Cancer Res.*, **47**, 1560.

ROWE, T.C., CHEN, G.L., HSIANG, Y.-H. & LIU, F. (1986). DNA damage by antitumour acridines mediated by mammalian DNA topoisomerase II. *Cancer Res.*, **46**, 2021.

SEELIG, G.F. & MEISTER, A. (1985). Glutathione biosynthesis: $\gamma$-glutamylcysteine synthetase from rate kidney. *Methods Enzymol.*, **113**, 379.

SEKURA, R.D., DUFFEL, M.W. & JAKOBY, W.B. (1981). Aryl sulfotransferases. *Methods Enzymol.*, **77**, 197.

SHEN, D.-W., FOJO, A., CHIN, J.E. & 4 others (1986). Human multidrug-resistant cell lines: increased *mdr* 1 expression can precede gene amplification. *Science*, **232**, 643.

SHOEMAKER, R.H., CURT, G.A. & CARNEY, D.N. (1983). Evidence for multidrug-resistant cells in human tumour cell populations. *Cancer Treat. Rep.*, **67**, 883.

SHOEMAKER, D.D., CYSYK, R.L., GORMLEY, P.E., DE SOUZA, J.J.V. & MALSPEIS, L. (1984). Metabolism of 4'-(9-acridinylamino)-methanesulfon-*m*-anisidide by rat liver microsomes. *Cancer Res.*, **44**, 1939.

SINHA, B.K., KATKI, A.G. & BATIST, G. (1987). Adriamycin-stimulated hydroxyl radical formation in human breast tumour cells. *Biochem. Pharmacol.*, **36**, 793.

SNOW, K. & JUDD, W. (1987). Heterogeneity of a human T-lymphoblastoid cell line. *Exp. Cell Res.*, **171**, 389.

STREETER, D.G., JOHL, J.S., GORDON, G.R. & PETERS, J.H. (1986). Uptake and retention of morpholinyl anthracyclines by adriamycin-sensitive and -resistant P388 cells. *Cancer Chemother. Pharmacol.*, **16**, 247.

STROBEL, H.W. & DIGNAM, J.D. (1978). Purification of NADPH-cytochrome P-450 reductase. *Methods Enzymol.*, **LII**, 89.

TSURUO, T. (1983). Reversal of acquired resistance to *Vinca*, alkaloids and anthracycline antibiotics. *Cancer Treat. Rep.*, **67**, 889.

VAN DER BLIEK, A.M., MEYERS, M.B., BIEDLER, J.L., HES, E. & BORST, P. (1986). A 22 kd protein (sorcin/V19) encoded by an amplified gene in multidrug-resistant cells, is homologous to the calcium-binding light chain of calpain. *EMBO J.*, **5**, 3201.

VASANTHAKUMAR, G. & AHMED, N.K. (1986). Contribution of drug transport and reductase to daunorubicin resistance in human myelocytic cells. *Cancer Chemother. Pharmacol.*, **18**, 105.

VOSBERG, H.P. (1985). DNA topoisomerases: enzymes that control DNA conformation. *Curr. Topics Microbiol. Immunol.*, **114**, 19.

WARHOLM, M., GUTHENBERG, C., VON BAHR, C. & MANNERVIK, B. (1985). Glutathione transferase from human liver. *Methods Enzymol.*, **113**, 499.

YANOVICH, S. & TAUB, R.N. (1983). Differences in daunomycin retention in sensitive and resistant P388 leukemic cells as determined by digitized video fluorescence microscopy. *Cancer Res.*, **43**, 4167.

ZWELLING, L.A. (1985). DNA topoisomerase II as a target of antineoplastic drug therapy. *Cancer Metast. Rev.*, **4**, 263.

ZWELLING, L.A., MICHAELS, S., ERICKSON, L.C,. UNGERLEIDER, R.S., NICHOLS, M. & KOHN, K.W. (1981). Protein-associated DNA strand breaks in L1210 cells treated with the DNA intercalating agents 4′-(9-acridinylamino)methane-sulfon-*m*-anisidide and adriamycin. *Biochemistry*, **20**, 6553.

ZWELLING, L.A., KERRIGAN, D., MICHAELS, S. & KOHN, K.W. (1982). Cooperative sequestration of mAMSA in L1210 cells. *Biochem. Pharmacol.*, **31**, 3269.